# Migratory behavior of eastern North Pacific gray whales tracked using a hydrophone array

**DOI:** 10.1371/journal.pone.0185585

**Published:** 2017-10-30

**Authors:** Regina A. Guazzo, Tyler A. Helble, Gerald L. D’Spain, David W. Weller, Sean M. Wiggins, John A. Hildebrand

**Affiliations:** 1 Scripps Institution of Oceanography, University of California San Diego, La Jolla, California, United States of America; 2 SPAWAR Systems Center Pacific, San Diego, California, United States of America; 3 National Oceanic and Atmospheric Administration, Southwest Fisheries Science Center, La Jolla, California, United States of America; Institute of Deep-sea Science and Engineering, Chinese Academy of Sciences, CHINA

## Abstract

Eastern North Pacific gray whales make one of the longest annual migrations of any mammal, traveling from their summer feeding areas in the Bering and Chukchi Seas to their wintering areas in the lagoons of Baja California, Mexico. Although a significant body of knowledge on gray whale biology and behavior exists, little is known about their vocal behavior while migrating. In this study, we used a sparse hydrophone array deployed offshore of central California to investigate how gray whales behave and use sound while migrating. We detected, localized, and tracked whales for one full migration season, a first for gray whales. We verified and localized 10,644 gray whale M3 calls and grouped them into 280 tracks. Results confirm that gray whales are acoustically active while migrating and their swimming and acoustic behavior changes on daily and seasonal time scales. The seasonal timing of the calls verifies the gray whale migration timing determined using other methods such as counts conducted by visual observers. The total number of calls and the percentage of calls that were part of a track changed significantly over both seasonal and daily time scales. An average calling rate of 5.7 calls/whale/day was observed, which is significantly greater than previously reported migration calling rates. We measured a mean speed of 1.6 m/s and quantified heading, direction, and water depth where tracks were located. Mean speed and water depth remained constant between night and day, but these quantities had greater variation at night. Gray whales produce M3 calls with a root mean square source level of 156.9 dB re 1 *μ*Pa at 1 m. Quantities describing call characteristics were variable and dependent on site-specific propagation characteristics.

## Introduction

Eastern North Pacific gray whales (*Eschrichtius robustus* Lilljeborg) undertake one of the longest migrations of any mammal, covering 50 degrees or more of latitude and traveling some 15,000 to 20,000 kilometers roundtrip between Baja California, Mexico and the Bering and Chukchi Seas [1]. By late November, most gray whales in the eastern North Pacific population are moving south from their summer feeding areas in the Bering and Chukchi Seas to wintering areas off Baja California, Mexico [2]. The gray whale migration is segregated by age, sex, and reproductive condition [3]. The first pulse of migrants is led by (a) near-term pregnant females, followed by (b) estrous females and mature males, and then (c) immature animals of both sexes [3]. The northward migration is segmented into two phases. The first phase includes (a) newly pregnant females, followed later by (b) adult males and anestrous females, and then (c) immature whales of both sexes [4]. The second phase consists mostly of mothers with calves that are observed on the migration route between March and May [4–6] and generally arrive to the summer feeding areas between May and June [3, 7]. Animals migrating southward tend to travel within five kilometers from shore [8, 9] while the northward migration is slightly more offshore and direct for most whales, except for cows with calves who keep within 200–400 m from shore in some areas [4, 5]. This migration pattern, hugging the North American coast, makes the eastern North Pacific gray whale migration the most observed whale migration, and also makes these whales more likely to be impacted by nearshore human activities.

The eastern North Pacific population of gray whales is often cited as a conservation success story. When commercial whaling first began, the eastern North Pacific gray whale population size was around 30,000 [10]. Between 1846 and 1874, whalers killed approximately 10,800 gray whales, causing the population to shift its migration corridor farther offshore and nearly desert the Mexican breeding lagoons [10]. In the 20th century, whaling on gray whales decreased with 2,724 whales taken from the eastern population between 1910 and 1946 [7]. In the 1950s and 1960s, aerial and land-based censuses estimated the population size to be 2,894–4,454 individuals [8, 11]. Today, eastern North Pacific gray whales are presumed to be at their carrying capacity [12] and are listed as a species of least concern [13]. The population size was estimated to be 28,790 individuals for the 2014–2015 season [14]. The dramatic recovery in population size over fifty years and the reoccupation of the lagoons in the winter and the coastal corridor during the migration gives hope that endangered marine mammals can survive with proper management and time.

The abundance of gray whales has been estimated by the National Oceanic and Atmospheric Administration (NOAA) Southwest Fisheries Science Center (SWFSC) with shore-based visual surveys during the southbound migration from December through February since the 1967–1968 migration season [15]. The visual observers for these surveys count pods and estimate the number of whales in each from a vantage point at or near Granite Canyon, south of Monterey, California, approximately 20 m above sea level [15, 16]. Observations are limited to daylight periods, suitable environmental conditions, and, in part, the ability to track and record multiple whales simultaneously migrating past the observation station. NOAA SWFSC added an infrared camera system in 2014–2015 that can detect whales based on the heat difference between their warm blows and the backdrop of the cool ocean during both the day and night.

Many studies have quantitatively described the gray whale migration by visually observing whales or satellite tagging a small sample. These studies show that most pregnant females travel south alone, but small, unstable groups of two or three are most common for the rest of the southbound migration [3, 8]. Migrating gray whales move steadily in one direction, breathing and diving in predictable patterns and generally swim at speeds between 1.1 and 2.8 m/s [1, 3, 17–21]. In order to minimize their cost of transport, gray whales should spend most of their time at depths below 2.5 times their maximum body width, or 6–7 m deep for an adult gray whale [7]. Most do not engage in other activities besides traveling although some later southbound gray whales display courting and mating behavior [8]. Acoustic monitoring using hydrophone arrays, as reported herein, offers an additional method to investigate migratory timing and behavior and provides data day and night and in all types of weather conditions.

Controversy exists regarding gray whale behavior at night because it is difficult to observe. Hubbs and Hubbs reported that gray whales continue migrating in bright moonlight, but stop on dark nights [11]. Perryman et al. [19] used infrared cameras for portions of three southbound migration seasons and observed that gray whales shifted 0.4 km closer to shore at night. In addition, after 15 January, gray whales increased their southbound migration rate at night and increased their pod size during the day [19]. Perryman et al. [19] hypothesized that gray whales were socializing more during the day and slowing their migration rate. A diel change in migratory behavior has not been observed during the northbound migration [5, 20]. These results warrant further investigation and since gray whales are known to make underwater sounds, acoustic monitoring provides another way to increase understanding of whale behavior during both night and day.

The acoustic behavior and role that vocalizing may serve during the gray whale migration is poorly understood. Dalheim [22] named and described six gray whale call types from sounds recorded in the Baja California lagoons. She recorded 0.33 calls/hour/whale in February and 0.25 calls/hour/whale in March and found no diel variation in call production [22]. Gray whales are reported to produce fewer calls while migrating than while in their breeding areas. Crane and Lashkari [23] measured 0.050 calls/hour/whale in water less than 100 meters deep and Cummings et al. [17] recorded 0.74 calls/hour with a maximum of 53 calls/hour/whale and noted that approximately one-third of solitary gray whales made detectable sounds during migration.

The M3 call is the most common call while migrating and makes up 47 to 87% of the total calls [17, 23]. The M3 call is an amplitude- and frequency-modulated call often containing harmonics [23]. It has been previously reported to have a peak frequency (which corresponds to Crane and Lashkari’s “center frequency”) below 100 Hz [23], a bandwidth from 20–200 Hz, and an average duration of 1.54 s [17]. The gray whale M3 calls have a reported source level of 151 dB re 1 *μ*Pa at 1 m off San Diego [17], but source levels of 167 to 188 dB re 1 *μ*Pa at 1 m have been reported for all gray whale call types in the Chukchi Sea [24]. The metric of the received call used to estimate source level (e.g. root mean square, 0-to-peak, peak-to-peak) was not reported in these published studies.

Another call type produced by migrating gray whales is the M1 call and makes up about 37% of the total calls [23]. This call is described to sound like knocking, metallic pulsing, or bongo drums [17, 23, 25]. M1 pulses are broadband calls containing an average of five pulses per call with the lowest frequency below 100 Hz and the highest frequency over 10 kHz [23, 25]. The M1 calls have a reported source level of 141 dB re 1 *μ*Pa at 1 m [17].

Earlier studies on the acoustic behavior of gray whales during migration have relied on relatively small sample sizes recorded over short time periods [17, 23]. The aim in this study was to use passive acoustic recorders offshore of the Granite Canyon visual survey site to monitor the gray whale migration and localize gray whale calls over a full migration season. In this way, we developed an understanding of their underwater movement and vocalizations with a large sample size and investigated whether this behavior changed over the migration cycle. We show that gray whales are acoustically active when migrating and their behavior changes over seasonal and daily time scales.

## Materials and methods

**Fig 1:**
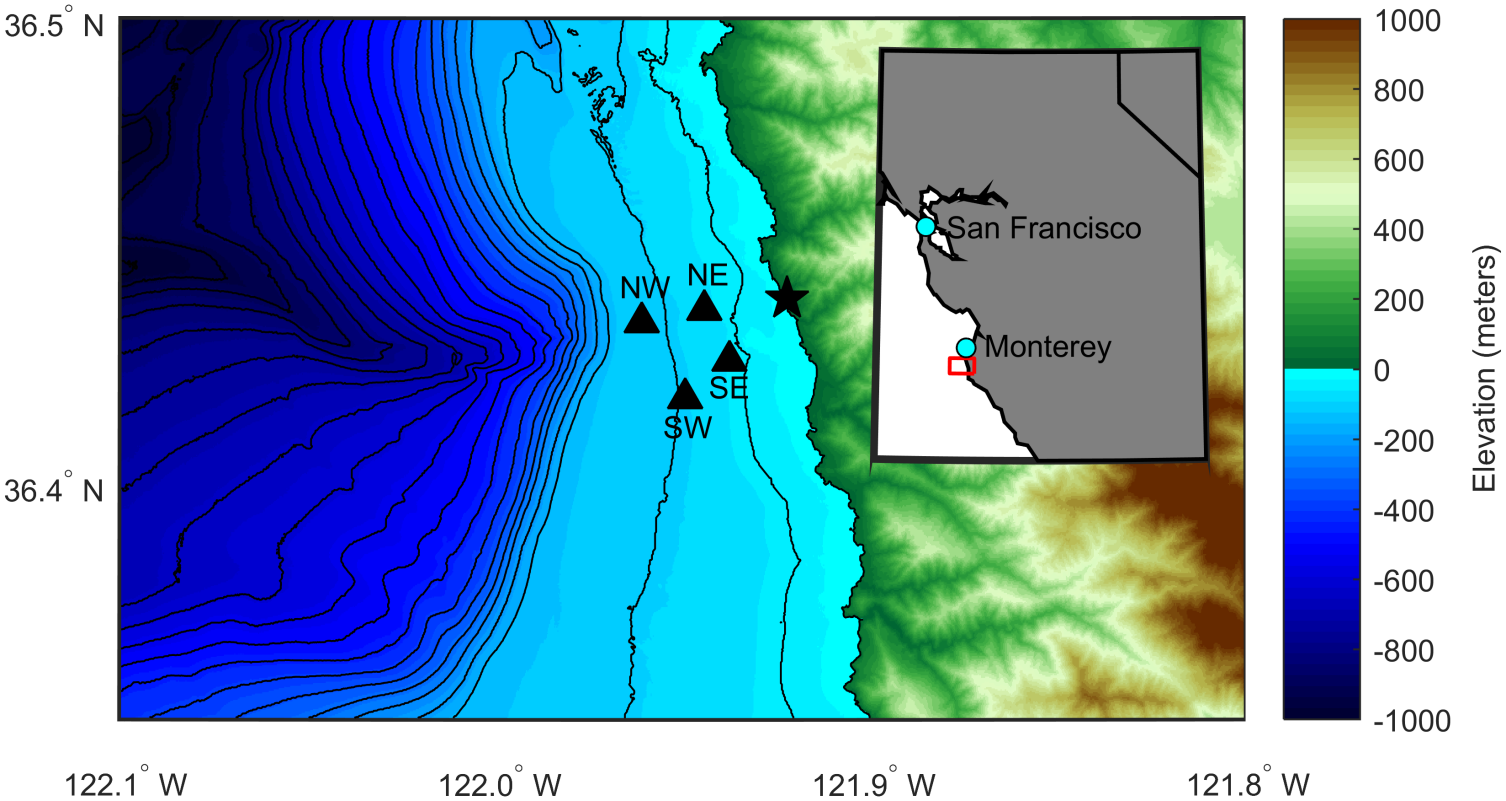
Deployment locations of acoustic recording packages. The study area is off central California as denoted by the red box in the inset map. Acoustic recording packages are indicated with black triangles and labeled according to their relative positions. The location of the NOAA Southwest Fisheries Science Center surveys are indicated with a black star. Colors indicate land elevation and seafloor depth with respect to sea level. Black contour lines show seafloor depth in 50 m increments. Bathymetry data from the NOAA National Centers for Environmental Information’s Southern California Coastal Relief Model with 1 arc-second resolution.

We deployed four acoustic recording packages offshore of the NOAA SWFSC survey site at Granite Canyon (Fig 1) under the Monterey Bay National Marine Sanctuary Research permit MBNMS-2014-039. Each acoustic recording package mooring consisted of a hydrophone, a datalogger, subsurface floats, an acoustic release, and weights for anchor (S1 Fig). They are based on Scripps Institution of Oceanography’s High-frequency Recording Package (HARP) [26]. These recording systems were bottom-moored systems that sampled at 2,000 Hz for an effective bandwidth from 10 to 1,000 Hz and recorded continuously from November 2014 until June 2015. The depths and locations of each acoustic recording package are listed in Table 1 and each hydrophone is denoted by its relative position as NE, NW, SE, or SW. The locations were chosen to surround the visually-determined main gray whale migration corridor 2 to 3 km from shore and to record the same whales detected during the NOAA surveys.

**Table 1:** Deployment locations of acoustic recording packages.

Hydrophone	Latitude	Longitude	Depth	Range to NE
NE	36.43778°N	121.94417°W	67	—
NW	36.43512°N	121.96082°W	110	1,530
SE	36.42679°N	121.93743°W	58	1,380
SW	36.41872°N	121.94925°W	94	2,170

Hydrophones are named according to their relative positions. Depth is water depth in m. Range is horizontal distance to the NE hydrophone in m. The hydrophone in each recording package was moored 15 m off the bottom in all 4 cases.

After recovery, all audio files were time-aligned by assuming linear clock drift across the deployment period. The clock drifts over the 7-month period were 0.1527, 2.0287, 0.5790, and 14.3578 s for the NE, NW, SE, and SW hydrophones, respectively. The least squared differences of measured time delays from theoretical time delays for localized calls were plotted and the values had a mean close to zero over the entire deployment indicating that linear clock drift was a valid approximation.

To detect gray whale vocalizations, we used the Generalized Power Law (GPL) Detector described by Helble et al. [27] and modified the parameter file for gray whale detections using a frequency band of 20–100 Hz, call length of 1.2–5 s, and fast Fourier transform (FFT) length of 1024 samples with 95% overlap. The GPL detector was run on the data output from all four hydrophones separately. All gray whale M3 call detections from the NE hydrophone recordings were verified manually.

To localize a gray whale call, a detection must be made on all four hydrophones (only three hydrophones are needed to localize, but we require four to increase precision). Helble et al. [28] described these localization methods in full detail with humpback whale vocalizations on the U.S. Navy’s Pacific Missile Range Facility. Since gray whales call less frequently than humpback whales, single calls were cross-correlated instead of a series of calls. Call spectrograms were normalized in each frequency bin based on the average noise level and the background noise was then set to zero to create a template (as in Section IIA of [28] and Eqs 10,11 in [27]). Background noise was defined as less than 0.5 times the average noise across all frequency bins and time (as opposed to 5 times the noise in [28]). Each template was cross-correlated with the template of the same call received on the NE hydrophone to calculate the time delay or time difference of arrival (TDOA) (similar to Section IIB in [28], except with cross-correlating single calls). Three TDOA measurements were obtained for each localized detection. The peak of each cross-correlation was made more precise using polynomial interpolation between the maximum cross-correlation values.

To obtain theoretical TDOA values, whales were assumed to be calling at 10 m depth, but since the water depth is an order of magnitude shallower than the spacing of the recording array, the assumed calling depth of the whale is insignificant. We verified whether changing the assumed depth of the whale would change the locations and we did not observe significant changes over the tested depths. On average, changing the assumed calling depth by 40 m (to 50 m deep) changes the estimated horizontal location of the animal by only 15 m, which is a small difference compared to range over which we are localizing.

No data on the sound speed profile in Granite Canyon were collected during the recording period and online oceanographic data bases contain few measurements near the recording site for the time period of data recording. Therefore, a constant sound speed of 1500 m/s was used in this analysis. For calls with primarily low frequency content recorded in this shallow water environment, the results should be insensitive to the sound speed profile details. An alternate sound speed profile of 1,490 m/s was tested, but no significant difference in call localizations were observed. Since the southbound migration peaks in January and the northbound migration peaks in March, we assume the sound speed profile will be about the same across the gray whale migration period. Any differences between the actual sound speed profile and these values will cause a bias in the localizations of calls.

A grid search method with 0.001° resolution over a search area of 36.40°–36.45°N and 121.92°—121.98°W determined the location of a calling whale based on the least-squared difference between the theoretical and measured TDOAs summed over the three TDOA measurements (according to Eq 1 in [28]). The whale location was refined by cubic interpolation of the sum of the least-squared difference around the location of the minimum.

Localized calls were then grouped into tracks. We developed a graphical user interface that allowed the user to slowly advance in time through the localized detections plotted on a map of the search area and select times with one or more tracks. To be considered valid, a track must consist of at least five calls. Each track was then automatically segmented based on maximum allowable time intervals, distance, and speed between sequential localizations. Since gray whales often travel in groups, this method allowed us to more easily remove calls that did not fit with the rest of the track and therefore may have been produced by another whale. Occasionally some calls in a track may be produced by another animal in a tight-knit group, but we are assuming that the majority of calls are produced by the focal animal and all track metrics are valid for that animal.

After tracks were defined, we plotted spectrograms of all calls in each track and verified call types and species. In addition to gray whales, tracks of humpback, fin, and blue whales were recorded by the hydrophone array.

Swimming behavior of calling gray whales was quantified by calculating speed and direction of tracks and noting seafloor depth at the track location. A smoothing spline was fit between the localizations in each track. Using a smooth curve as a track better models actual swimming behavior instead of connecting each localization with a straight line. Average speed, heading, and direction index for each track was then calculated. Southbound whales are defined as any track with a heading between +135° and +225° and northbound whales are defined as any track with a heading between -45° and +45°. Direction index is a metric developed to measure how straight or curved a track is and is calculated by dividing the net distance traveled by the total distance traveled. A direction index of one indicates a straight track while an index of zero indicates no net movement in location. The seafloor depth along each track was also recorded. Bathymetry data were retrieved from the NOAA National Centers for Environmental Information using the Southern California Coastal Relief Model with 1 arc-second resolution (https://maps.ngdc.noaa.gov/viewers/wcs-client/). These metrics were compared over seasonal and daily time scales.

Not all calls that were localized contributed to a track. A whale had to call at least five times as it swam through the search area and those calls must have been detected on all four hydrophones to be categorized as a track. M1 calls were not manually verified for localization since few are detected on all four hydrophones. Since the majority of gray whale calls detected were M3, using the percentage of M3 calls that were part of a track is a valid metric to analyze how calling behavior changes over different time scales. Although M1 calls were not enumerated for total call counts, some tracks with M3 calls also had M1 calls.

Since whales swimming past the study location have a well-defined spatial distribution and abundance from visual observations, we are able to calculate an average population calling rate over the entire deployment. When a whale vocalized an M3 call within the area of the hydrophone array, it had a very high probability of detection and localization. Only calls within the area bounded by the array were used in this calling rate analysis. From aerial surveys at Granite Canyon, it is known that approximately 95% of gray whales migrate south within 4.17 km of shore [9]. The eastern-most hydrophone in this study was approximately 1.7 km from shore and the western-most hydrophone was approximately 3.4 km from shore. Since we are not counting the small fraction of whales that travel inshore and offshore of the hydrophone array, we assumed that 90% travel through the array. We used the 2014–2015 abundance estimate of 28,790 whales [14]. Travel time was estimated as the total distance traveled to pass through the array divided by the speed of the whale. Calling rate was calculated by dividing the total calls by the product of the total whales and the travel time. Population calling rate has units of calls/whale/day.

To expand on the description of received call types presented in past gray whale acoustic studies, we measured several aspects of the received calls. For all M3 calls produced within the area of the hydrophone array, we measured the received call duration, peak frequency, 3 dB bandwidth, mean frequency, and ±*σ* bandwidth. A full description of peak frequency (referred to in Crane and Lashkari as center frequency) and 3 dB bandwidth is given in Crane and Lashkari [23]. Peak frequency is the frequency with the greatest amplitude in the call spectrum and the 3 dB bandwidth is a measure of the bandwidth of that peak frequency (bandwidth of a single harmonic). The 3 dB bandwidth metric was estimated using linear extrapolation from the peak frequency. Mean frequency is a weighted mean where the weighting is determined by the fraction of total energy at each frequency over the bandwidth of the call. That is, if *A*^2^(*f*_*i*_) is the magnitude squared in the i-th frequency bin centered at frequency *f*_*i*_, then

$$\begin{array}{c} \mu \left(f\right)=\sum_{i=f_{1}}^{f_{2}}w_{i}f_{i} \end{array}$$ (1)

where the weighting is

$$\begin{array}{c} w_{i}=\frac{A^{2}\left(f_{i}\right)}{\sum_{i=f_{1}}^{f_{2}}A^{2}\left(f_{i}\right)} \end{array}$$ (2)

and *f*_1_ is 20 Hz and *f*_2_ is 200 Hz for M3 calls and 1,000 Hz for M1 calls. The ±*σ* bandwidth can be defined as ±1 standard deviation about the mean frequency. The variance is

$$\begin{array}{c} \sigma^{2}\left(f\right)=\sum_{i=f_{1}}^{f_{2}}w_{i}{\left(f_{i}-\mu \left(f\right)\right)}^{2} \end{array}$$ (3)

and so the ±*σ* bandwidth is $2\sqrt{\sigma^{2}}$. The hydrophones had a roll-off in sensitivity and recorded high noise below 20 Hz so only frequencies greater than 20 Hz were used in spectral analysis. The upper frequency limit was chosen based on the call characteristics as visualized with spectrograms.

To separate the component of variance of a given call characteristic associated with variation in the environmental properties across the four elements of the array from the total variance, we calculated the variance of the call characteristics three different ways. First we define *a*_*i*,*j*_ as some characteristic of the *i*^*th*^ call recorded by the *j*^*th*^ hydrophone. The component of the variance that is comprised primarily of the variance due to variations from one call to the next is calculated by taking the mean of the measurements across all four hydrophones and then calculating the variance of that mean (referred to as *SD*^2^)

$$\begin{array}{c} SD^{2}=\frac{1}{N}\sum_{i=1}^{N}{\left(\bar{a_{i}}-<\bar{a}>\right)}^{2} \end{array}$$ (4)

where $\bar{a_{i}}$ is the mean of the characteristics of a single call over all four hydrophones or

$$\begin{array}{c} \bar{a_{i}}=\frac{1}{4}\sum_{j=1}^{4}a_{i,j} \end{array}$$ (5)

and the mean over all calls and hydrophone recordings is

$$\begin{array}{c} <\bar{a}>=\frac{1}{N}\sum_{i=1}^{N}\bar{a_{i}}=\frac{1}{4N}\sum_{i=1}^{N}\sum_{j=1}^{4}a_{i,j} \end{array}$$ (6)

The component primarily associated with the environment is calculated by taking the variance of the measurements for each call across the four hydrophones and then taking the mean of these variances for all the calls (referred to as $SD_{per\;call}^{2}$)

$$\begin{array}{c} SD_{per\;call}^{2}=\frac{1}{N}\sum_{i=1}^{N}(\frac{1}{4}\sum_{j=1}^{4}{\left(a_{i,j}-\bar{a_{i}}\right)}^{2}) \end{array}$$ (7)

The sum of these variances, i.e., the squares of the corresponding standard deviations, equal the total population variance ($SD_{all}^{2}$)

$$\begin{array}{c} SD^{2}+SD_{per\;call}^{2}=SD_{all}^{2} \end{array}$$ (8)

where

$$\begin{array}{c} SD_{all}^{2}=\frac{1}{4N}\sum_{i=1}^{N}\sum_{j=1}^{4}{\left(a_{i,j}-<\bar{a}>\right)}^{2} \end{array}$$ (9)

In summary, the individual quantities can be interpreted as:

1. *SD*^2^: The variance of the means of the calls, where the means are calculated across all four hydrophones. This variance is the variance in received characteristics over all calls.
2. $SD_{per\;call}^{2}$: The mean of the variance of a given call across all four hydrophones, where the mean is calculated over all recorded calls. This variance is the variance due to site-specific effects such as those caused by variations in propagation or variations in the properties of the individual data acquisition systems. For those call characteristics used in time-of-arrival difference estimation, it provides a quantitative measure of the effects of site-specific variations in localization.
3. $SD_{all}^{2}$: The total population variance. This variance is the variance over all call recordings by all hydrophones.

M1 calls were rarely detected on all four hydrophones. Since the sample size was only 23 for M1 calls detected within the array with good signal-to-noise ratios on all four hydrophones, analyst-detected M1 calls from a single hydrophone (NE) were used instead. We quantified inter-pulse interval (IPI), peak frequency, 3 dB bandwidth, mean frequency, and ±*σ* bandwidth for these M1 calls. The variance of IPI was compared both within a single call $SD_{w/in\;call}^{2}$) and between all calls (*SD*^2^).

Sound pressure spectral level calculations are used for estimating both received level and noise level. Spectral level integrated across a frequency bandwidth of interest is calculated by

$$\begin{array}{c} RL=\frac{f_{s}}{nFFT}*\sum_{i=f_{1}}^{f_{n}}Sp\left(f_{i}\right) \end{array}$$ (10)

where RL is the mean square received level, nFFT is the number of samples used in each FFT window, and the spectral density *Sp*(*f*_*i*_) is

$$\begin{array}{c} Sp\left(f_{i}\right)=2*\frac{1}{nT}\sum_{j=1}^{nT}\frac{{\left|X_{j}\left(f_{i}\right)\right|}^{2}}{f_{s}*nFFT*\left(\frac{1}{nFFT}*\sum_{i=1}^{nFFT}w_{i}^{2}\right)} \end{array}$$ (11)

where the subscript j indicates the j-th time segment and nT is the number of time segments incoherently averaged to obtain the spectral density estimate. The factor of 2 leading the right-hand side of the equation above accounts for the energy at negative frequencies. For gray whale M3 calls, *Sp*(*f*_*i*_) is summed from *f*_1_ = 20 Hz to *f*_*n*_ = 100 Hz. The quantity *X*_*j*_(*f*_*i*_) is the fast Fourier transformed complex value in the frequency bin corressponding to *f*_*i*_. The sum of $w_{i}^{2}$ is the sum of the square of all the points in the window applied to each of the j time series segments before Fourier transforming. We used a Hamming window for this analysis. Dividing by the ratio of the sampling frequency and the FFT length normalizes by the bin width in order to estimate spectral density. This step is necessary for estimating continuous spectra. Ocean noise typically has a continuous spectrum and since the M3 call has energy that spans several frequency bins, we treated it as a continuous spectrum and calculated spectral density. To convert into decibel units, we took 10 * log_10_(*RL*) ≡ *RL*_*dB*_. This method calculates the root mean square (RMS) received level, equivalent in the decibel domain to mean square amplitude.

Knowing the source level of a call is important for understanding how far away a call can be detected and how this detection range would change with changing background noise and acoustic propagation conditions. To estimate source level, we first measured received level of all calls localized within the area bounded by the hydrophones of the array. Since localization precision decreases with distance from the array, we only used calls within the array for this analysis. We subtracted the background noise from the spectrum to obtain the signal level without the noise and used the formulas above. At ranges from the source to the hydrophone greater than the seafloor depth at the source location, source level was estimated from received level by

$$\begin{array}{c} SL_{dB}=RL_{dB}+20*\log_{10}\left(r_{T}/1m\right)+10*\log_{10}\left(r/r_{T}\right)+\alpha *\left(r-r_{T}\right) \end{array}$$ (12)

where *SL*_*dB*_ is source level in decibel units, *RL*_*dB*_ is received level in decibel units, *r*_*T*_ is the transition range at which geometrical spreading transitions from spherical to cylindrical, *α* is the empirically determined attenuation/absorption coefficient due to bottom interaction, and r is the horizontal distance from the whale to the hydrophone [29]. At ranges less than the seafloor depth, source level was calculated using spherical spreading only over the slant range

$$\begin{array}{c} SL_{dB}=RL_{dB}+20*\log_{10}\left(r/1m\right) \end{array}$$ (13)

These equations are approximately derived by incoherently averaging TL over range and frequency. They assume homogenity and isotropy in the acoustic propagation conditions.

For the same call (i.e. the same SL) recorded at two different receivers at ranges *r*_1_ and *r*_2_ where *r*_1_ ≥ *r*_*T*_ and *r*_2_ ≥ *r*_*T*_ and assuming the source radiation pattern is omnidirectional

$$\begin{array}{c} RL_{2}-RL_{1}=10*\log_{10}\left(r_{1}/r_{2}\right)+\alpha *\left(r_{1}-r_{2}\right) \end{array}$$ (14)

If *r*_1_ ≥ *r*_*T*_ but *r*_2_ ≤ *r*_*T*_

$$\begin{array}{c} RL_{2}-RL_{1}=10*\log_{10}\left(r_{T}*r_{1}/r_{2}^{2}\right)+\alpha *\left(r_{1}-r_{T}\right) \end{array}$$ (15)

This equation can be solved for *r*_*T*_ using the empirically-derived value for *α* obtained from Eq 14.

Unfortunately, only three calls occurred where the range to one of the hydrophones was less than the water depth. Therefore, data from only nine pairs of hydrophones were available to empirically estimate *r*_*T*_, too small a sample size for a reliable estimate. Instead, we assumed spherical spreading from the location of the source to the seafloor. Since the assumed depth of the source is at 10 m and is therefore close to the surface, an estimated transition range (*r*_*T*_)(defined in [29]) of the seafloor depth at the source location (*D*_*src*_) was used. It is unknown at what depth or depths the whales are calling, although it is estimated that the whales spend most of their time around 6–7 m deep [7]. This assumption of a transition range of *D*_*src*_ affects the overall source level estimates somewhat. For example, using values of *r*_*T*_ of *D*_*src*_ and *D*_*src*_/2 results in differences in transmission loss of 10 * log_10_(2) − *α* * *D*_*src*_/2, or about 3 dB. Therefore, the use of *D*_*src*_ as the transition range may over-estimate the transmission loss and the resulting source level estimate by up to 3 dB.

For each call, there were 6 pairs of hydrophones from which to estimate the attenuation/absorption coefficient *α*. To protect against outliers, we took the median of the pairwise *α* calculations for each call. We then used the mean of *α* for all calls to estimate the RMS source level.

Source sound exposure level (SEL) was calculated from the RMS source level

$$\begin{array}{c} SEL=SL_{RMS}+10*\log_{10}\left(t_{call}\right) \end{array}$$ (16)

where *t*_*call*_ is the call duration in seconds. Finally peak-to-peak source level was calculated in the same way as RMS source level. Peak-to-peak level is the difference between the time series maximum and minimum no matter where in the call the maximum and minimum occur.

We estimated the sound pressure spectral level of the background noise to ensure that changes in numbers of calls were due to changes in animal behavior and not due to changes in probability of detection [30]. Background noise was estimated on each hydrophone recording for each minute of recording and calculated using root median square over the same 20–100 Hz band. Root median square is the same as root mean square except calculated by taking the median spectral level across time instead of the mean. Root median square was used so as to not overly emphasize time periods with isolated high-level short-duration pulses from the instrument’s self-noise. We did not remove other marine mammal vocalizations from the background noise calculations because the presence of other calls can also influence the probability of detection.

We calculated probability of localization during each minute bin for a grid of locations within the area of the array. The passive sonar equation

$$\begin{array}{c} SNR_{dB}=SL_{dB}-TL_{dB}-NL_{dB} \end{array}$$ (17)

was used with SL randomly chosen from the distribution of real RMS source levels, the transmission loss (TL) from a source at each location in the grid to each hydrophone, and the noise level at each hydrophone in that minute. We repeated the random selection of SL values 100 times for each grid location. A minimum signal-to-noise ratio (SNR) of 0.5 dB was required for detection, determined by adding an M3 call to various levels of Gaussian noise. The probability of detection at a hydrophone was equal to the percent of time that a random source level call had a received SNR greater than or equal to 0.5 dB. We repeated these calculations for each hydrophone and then used the minimum probability of detection across the four hydrophones at each location. Much of the high noise was due to instrument cable strumming because of the shallow-water environment, so it was important to calculate the probability of detection on each of the hydrophones to determine which hydrophone was limiting the localization. To calculate the probability of localization at each time, we took the mean of the probability of localization across the area within the array. This method assumes even spatial distribution of whales across the area within the array. To correct for noise, the number of calls localized during each minute was divided by the probability of localization in that minute. However, if the probability of localization in a minute was less than 50%, we did not count any calls detected in that minute and categorized that as a time with no effort. We then divided the corrected call count for each day by the proportion of minutes with a probability of localization at least 50% to get a normalized daily call count within the area of the array. All detections localized within the area of the array were manually verified on all four hydrophones.

## Results

**Fig 2:**
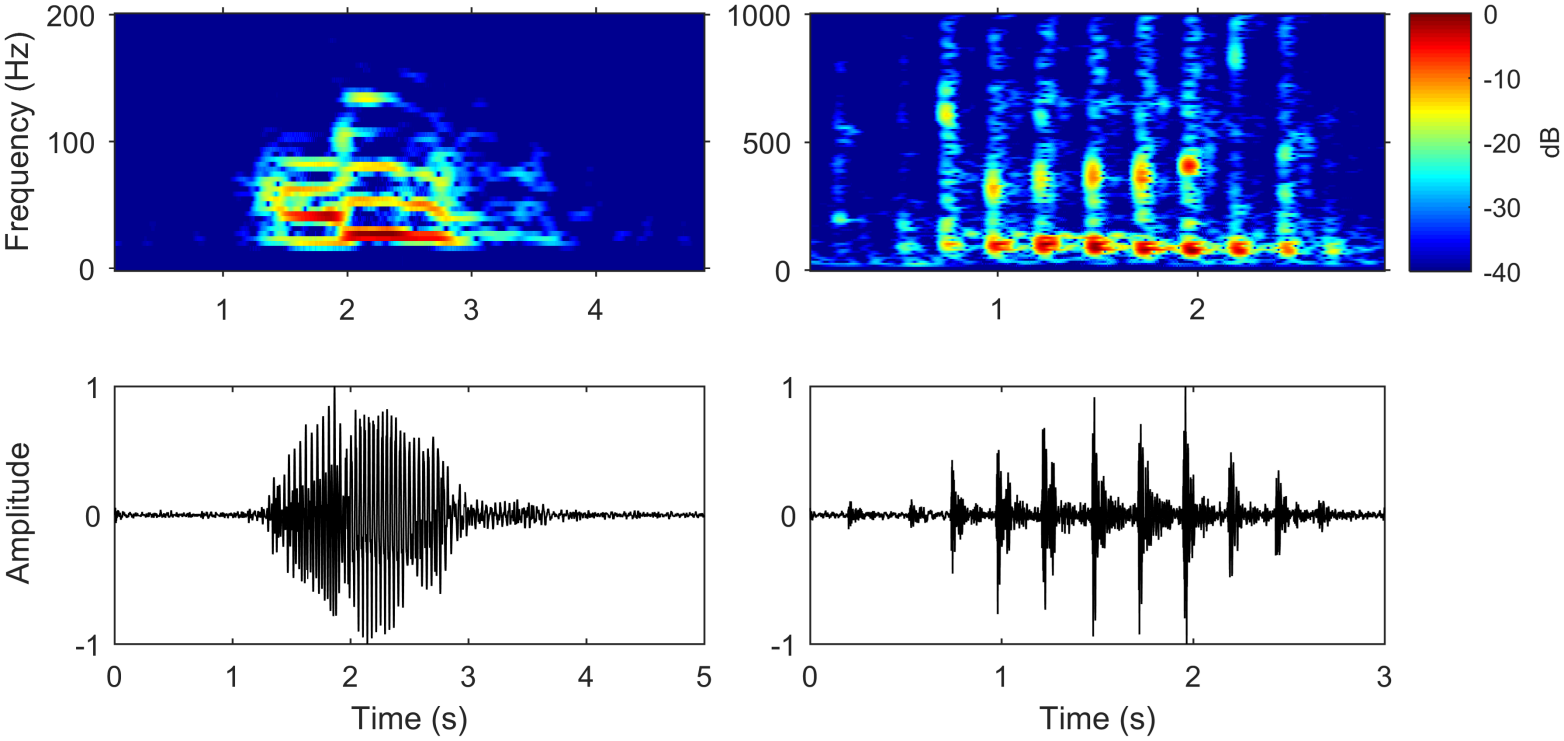
Gray whale M3 and M1 recorded vocalizations. An example (A) M3 call and (B) M1 call recorded on the NE hydrophone. Spectrograms are on top with frequency on the y-axis, time on the x-axis, and color indicating pressure magnitude squared (or equivalently pressure magnitude) in dB. A 40 dB dynamic range was used and all magnitudes were normalized to the greatest dB magnitude of the spectrogram. Note the different axes limits for the two call types. Time series plots are on the bottom with normalized amplitude on the y-axis and time on the x-axis. The M3 spectrogram has an FFT length of 512 with 99% overlap and a Hamming window and is bandpass filtered from 20 to 200 Hz. The M1 spectrogram has an FFT length of 256 with 99% overlap and a Hamming window and is bandpass filtered from 20 to 1,000 Hz.

In total, we detected, verified, and localized 10,644 gray whale M3 calls from 1 December 2014 to 3 May 2015. The majority of calls were M3. Fig 2 shows example time series and corresponding spectrograms of high signal-to-noise ratio M3 and M1 call types recorded on the NE hydrophone. Audio files of the pictured calls are provided in supplement files S1 and S2 Audio.

**Fig 3:**
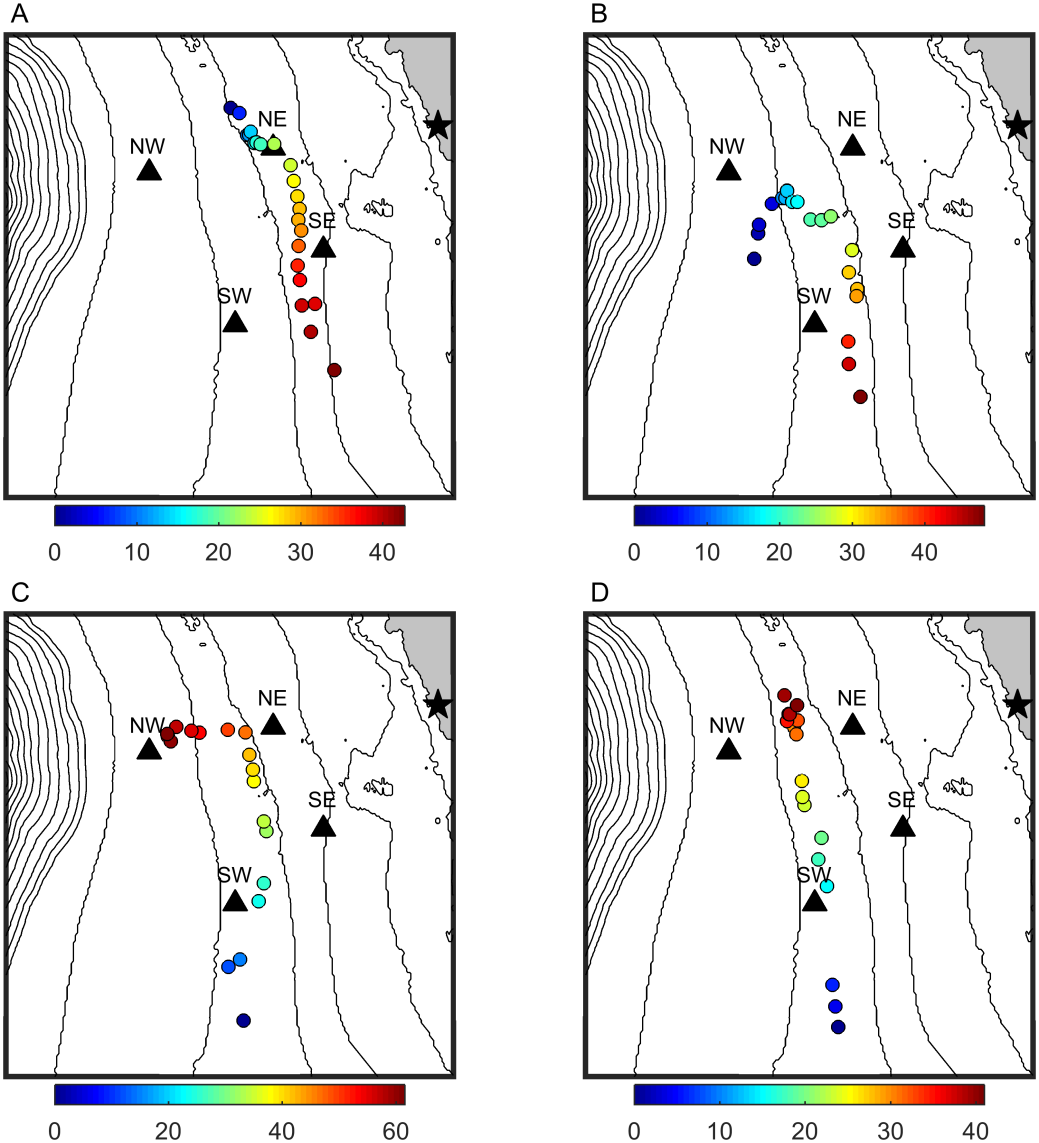
Examples of gray whale tracks from acoustic localization. Gray whale positions determined by localizing their vocalizations on the hydrophone array. Plots (A) and (B) show southbound whales (21 January 2015 and 10 February 2015, respectively) and plots (C) and (D) show northbound whales (13 March 2015 and 9 April 2015, respectively). Dots indicate position of the calling animal and their color matches the minutes since the start of the track with earlier in time in blue and later in red. The four black triangles mark the positions of the four bottom-mounted hydrophones and the black star marks the position of the NOAA visual and infrared camera research site. Contour lines show water depth in 20 m increments. The axes limits are 36.4° to 36.45° for latitude and -121.98° to -121.92° for longitude.

Gray whale calls were grouped into 280 tracks consisting of at least five calls with a total of 154 southbound gray whale tracks and 112 northbound gray whale tracks. The remaining 14 tracks did not have a clear northbound or southbound direction. Examples of four tracks are shown in Fig 3. Every detection that was part of a track was manually verified as being a gray whale call. The mean number of calls in the tracks (biased high because of the requirement that at least 5 calls define a track) was 8.4 and the maximum was 32. The mean of the mean inter-call interval in each track was 3.89 minutes with a standard deviation of 2.31 minutes. No trend was present in inter-call interval over time.

### Vocalization characteristics

Characteristics of M3 and M1 calls were quantified for calls with high signal-to-noise ratios. The received M3 call duration, peak frequency, 3 dB bandwidth, mean frequency, and ±*σ* bandwidth for 2,368 calls localized within the hydrophone array are presented in Table 2. The variance is separated by the amount attributable to variability from call to call (*SD*^2^) and the amount attributable to change on a single call during propagation ($SD_{per\;call}^{2}$). These values are also shown as percentages of the total variance for each measurement. It was not possible to discern between these two types of variance for M1 calls because the sample size of calls clearly detected on all four hydrophones was not adequate. The received M1 call inter-pulse interval, peak frequency, 3 dB bandwidth, mean frequency, and ±*σ* bandwidth for 190 calls consisting of at least four pulses detected on the NE hydrophone are presented in Table 3. The percentage of variance from changes in inter-pulse interval within a call is compared with the percentage of variance from changes in inter-pulse interval between calls. The frequencies chosen over which to integrate for mean frequency and ±*σ* bandwidth can dramatically influence the results so it is important that these are stated.

**Table 2:** Characteristics of received gray whale M3 calls.

M3	Signal Duration	Peak Frequency	3 dB Bandwidth	Mean Frequency	±*σ* Bandwidth
n = 2,368	(s)	(Hz)	(Hz)	(Hz)	(Hz)
**Mean**	1.79	38.1	4.17	48.1	44.9
***SD*^2^**	0.0460	228	9.36	88.6	95.3
$SD_{per\;call}^{2}$	0.0425	130	26.9	27.7	51.8
**% *SD*^2^**	52.0%	63.7%	25.8%	76.2%	64.8%
**%**$SD_{per\;call}^{2}$	48.0%	36.3%	74.2%	23.8%	35.2%

All calls were received and measured on all four hydrophones. These quantities are highly dependent on the site-specific propagation properties. *SD*^2^ is the variance of the mean values for each call and $SD_{per\;call}^{2}$ is the mean of the variances for each call across the four hydrophones. The relative contribution of each type of variance is shown as a percentage of the total variance.

**Table 3:** Characteristics of received gray whale M1 calls.

M1	Inter-Pulse Interval	Peak Frequency	3 dB Bandwidth	Mean Frequency	±*σ* Bandwidth
n = 190	(s)	(Hz)	(Hz)	(Hz)	(Hz)
**Mean**	0.208	149	8.07	377	556
***SD*^2^**	4.78 × 10^−3^	1.19 × 10^4^	35.7	8.14 × 10^3^	6.06 × 10^3^
$SD_{w/in\;call}^{2}$	4.62 × 10^−3^				
**% *SD*^2^**	50.8%				
**%** $SD_{w/in\;call}^{2}$	49.2%				

Calls were only measured on one (NE) hydrophone. *SD*^2^ is the variance of the mean values for each call and $SD_{w/in\;call}^{2}$ is the mean of the variances within each call. The relative contribution of each type of variance for inter-pulse interval is shown as a percentage of the total variance.

**Fig 4:**
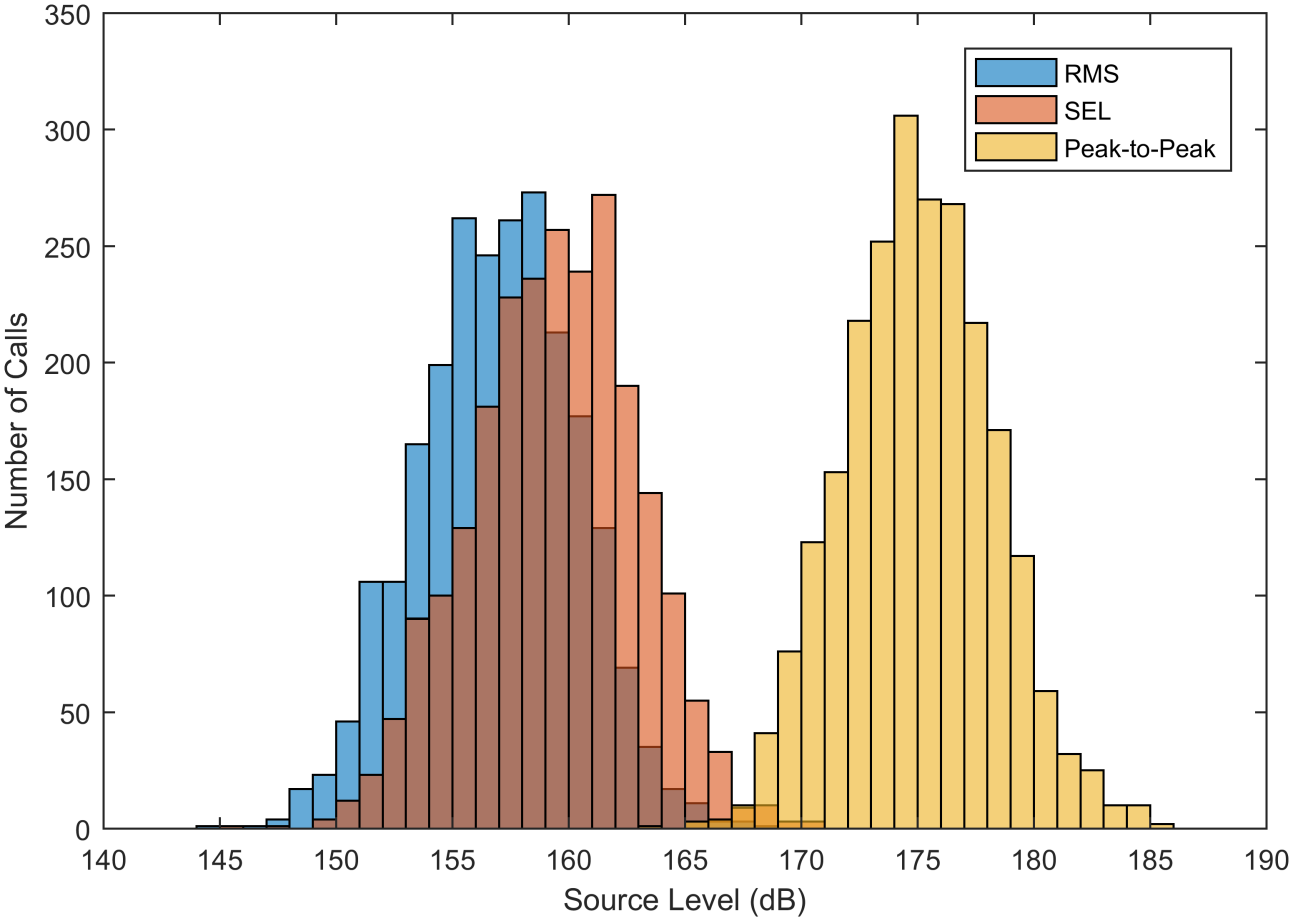
Estimated source level of gray whale M3 calls. These histograms show the source level of gray whale M3 calls. RMS is shown in blue (dB re 1 *μ*Pa at 1 m), SEL is shown in orange (dB re 1 *μ*Pa^2^ s at 1 m), and peak-to-peak is shown in yellow (dB re 1 *μ*Pa at 1 m).

Gray whale M3 calls have a mean RMS source level of 156.9 dB re 1 *μ*Pa at 1 m measured with bandwidth 20–100 Hz (n = 2,368 calls). The variance of the source level is 11.4 dB (calculated in the decibel domain). The attenuation/absorption coefficient *α* was estimated to be 4.26 × 10^−4^ which indicates that attenuation/absorption has little effect on the call and most transmission loss is due to spherical and cylindrical spreading. Estimated received level can vary by about 2 dB depending on the signal-to-noise ratio due to errors from subtracting the background noise from the signal level. This value was determined by inserting an M3 call in various levels of Gaussian noise. As a result, signals in high noise are reported as having a greater received level than signals in low noise. Means and variances of the calculated RMS, SEL, and peak-to-peak source levels are summarized in Table 4 and are plotted in Fig 4. Similar to Au et al. [31] with respect to humpback whale calls, we found that on average, gray whale M3 call peak-to-peak source level was 18.1 dB greater than RMS source level. SEL was 2.5 dB greater than RMS source level, which is what is expected from a mean call duration of 1.79 s.

**Table 4:** Source level of gray whale M3 calls.

	RMS (dB re 1 *μ*Pa)	SEL (dB re 1 *μ*Pa^2^ s)	Peak-to-Peak (dB re 1 *μ*Pa)
**Mean**	156.9	159.4	175.0
***SD*^2^**	11.4	12.4	10.4
$SD_{per\;call}^{2}$	6.82	6.94	6.56
**%*SD*^2^**	62.5%	64.1%	61.2%
**%** $SD_{per\;call}^{2}$	37.5%	35.9%	38.8%
**Mean** *α*	4.26 × 10^−4^	4.26 × 10^−4^	6.60 × 10^−5^
**Variance** *α*	3.37 × 10^−5^	3.37 × 10^−5^	2.67 × 10^−5^

The source level of gray whale M3 calls calculated three different ways. *SD*^2^ is the variance of the mean values for each call and $SD_{per\;call}^{2}$ is the mean of the variances for each call across the four hydrophones as in Table 2. Mean *α* is the environment-dependent attenuation/absorption coefficient and has units of dB/m. The mean and variance of *α* were calculated from the median for each call measured across all pairs of hydrophones. *α* is the same for RMS and SEL because the SEL was calculated from RMS. All means and variances were calculated in the dB domain.

### Seasonal cycle

#### Background noise and probability of localization

Noise level was calculated for each minute of the deployment on the NE hydrophone. The 25th, 50th, and 75th percentiles of root median square noise were 92.3, 93.5, and 94.9 dB re 1 *μ*Pa, respectively.

**Fig 5:**
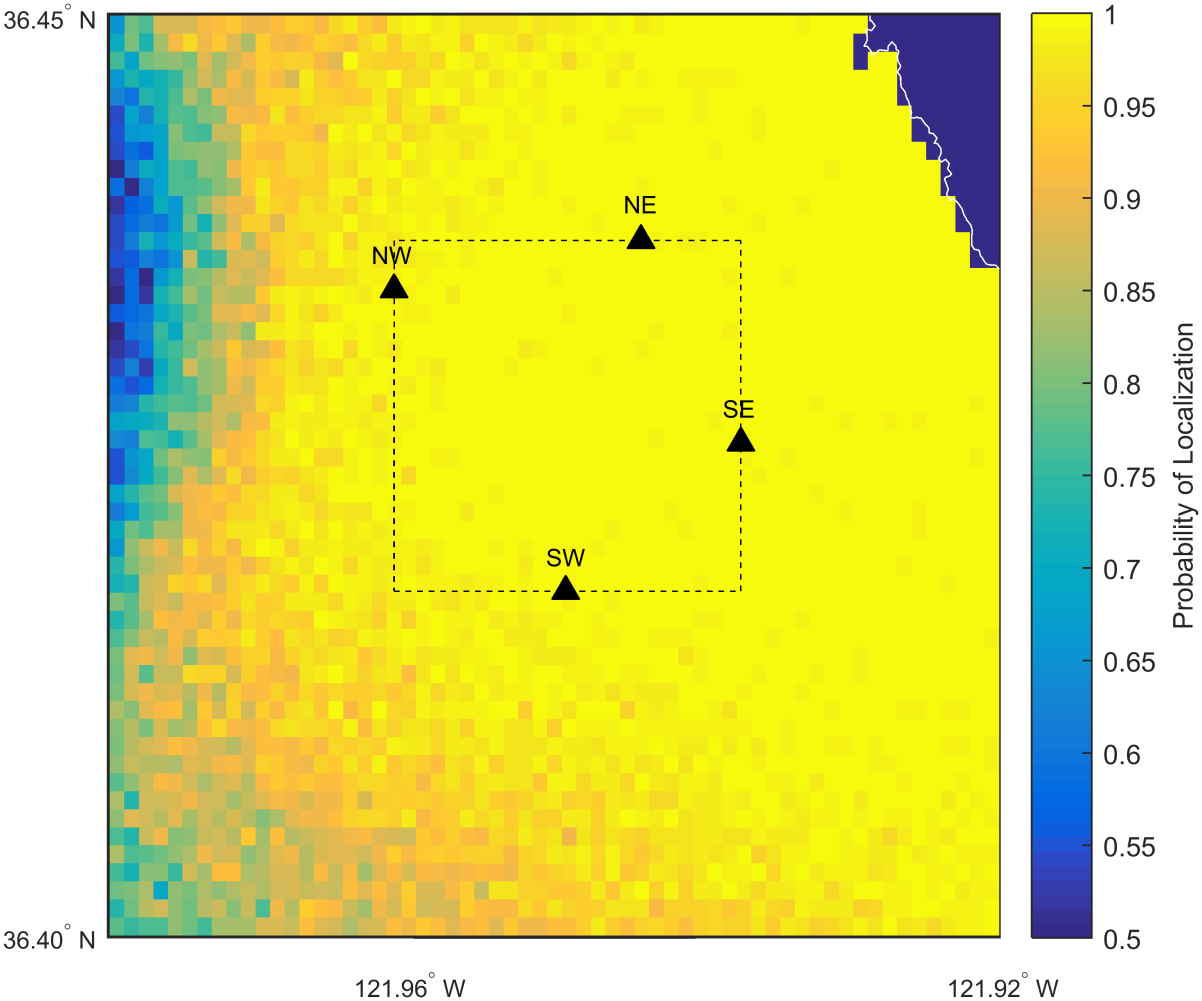
Probability of localization in 93.5 dB re 1 *μ*Pa root median square noise. This map shows the probability of localization of a call produced at locations close to the hydrophone array in 93.5 dB re 1 *μ*Pa root median square background noise. In the 50th percentile noise conditions, the probability of localization within the area of the array was approximately 100%. Probability of localization increases as noise levels decrease. The four black triangles mark the positions of the four bottom-mounted hydrophones. The dashed box indicates the area inside the hydrophone array. The axes limits are 36.4° to 36.45° for latitude and -121.98° to -121.92° for longitude.

At the median noise level of 93.5 dB re 1 *μ*Pa, the probability of localization within the array was approximately 100% (Fig 5). A total of 4,247 M3 calls were localized within the area of the array, but after correcting for the probability of localization, we estimate that 4,854 calls were produced within the area of the array. This call count is likely an underestimate because infrequent calling results in many minutes having zero calls and these values stay zero even after the noise correction. Only 10.1% of minutes over the entire deployment had a probability of localization below the 50% threshold. In a single day, the greatest percentage of the day considered to have no effort due to low probability of localization was 49%. High noise was most common during the first month of the deployment due to strumming of the acoustic recording package cables.

#### Total number of calls and percentage part of a track

**Fig 6:**
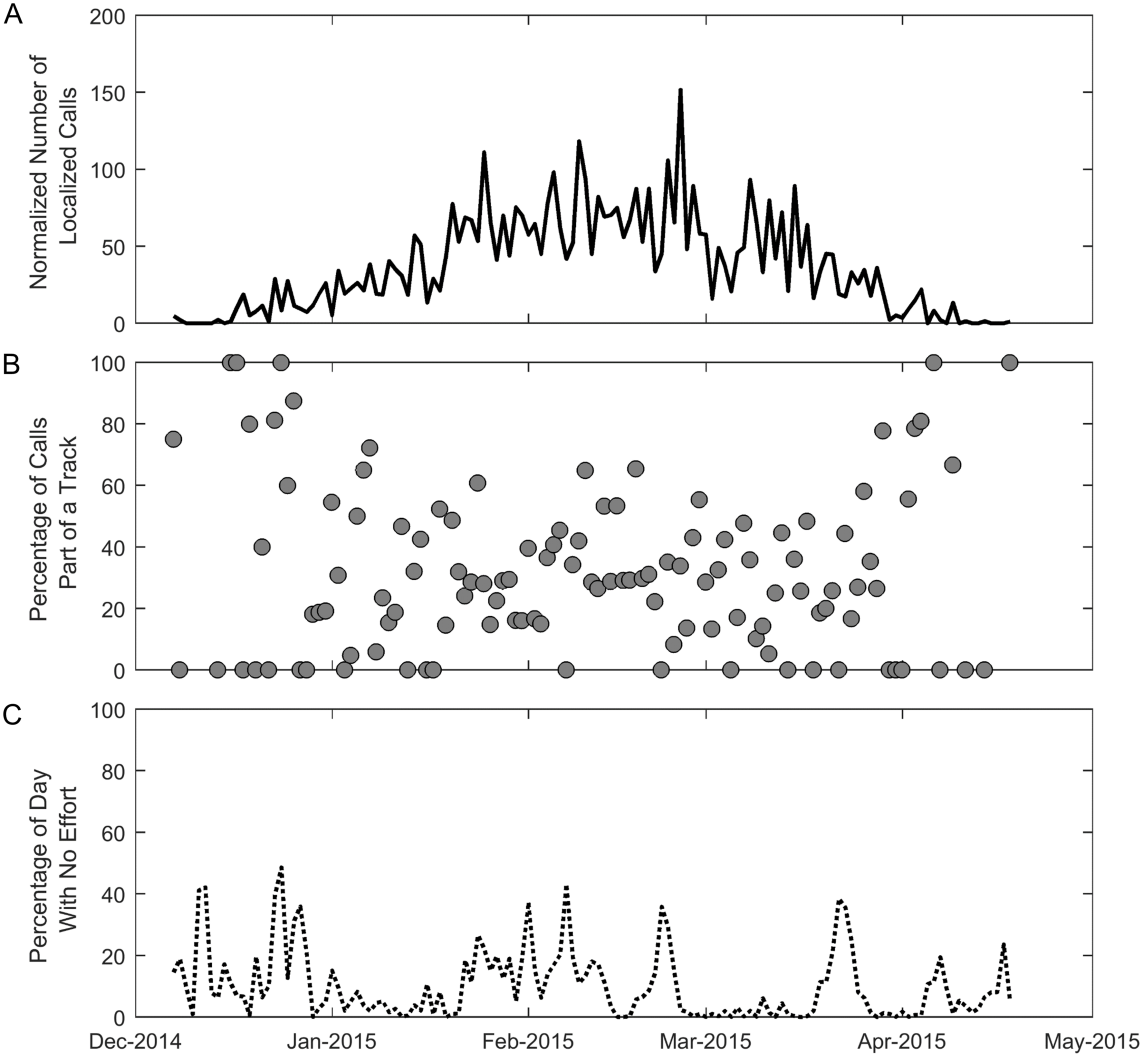
Seasonal cycle: Normalized calls and percentage of calls that were part of a track. The normalized number of localized calls per day is shown in (A), the percentage of those calls that were part of a track is shown in (B), and the percentage of the day with no effort due to probability of localization less than 50% is shown in (C). The first localized gray whale call within the area bounded by the array was detected on 7 December 2014 and the last was detected on 18 April 2015. All of the calls counted were manually verified on all four hydrophones to be gray whale M3 calls.

The total number of localized calls within the array per day increased from December 2014 until the middle of February with a maximum value of 139 localized calls (152 normalized calls) within the area bounded by the array on 25 February 2015 and then the total number of localized calls decreased until the middle of April (Fig 6). These calls were recorded on all four hydrophones and verified as M3 calls, but were not necessarily part of a track. The percentage of M3 calls within the array that were also part of a track had high variability, but in general a higher percentage of calls were part of a track at the beginning and end of the migration (Fig 6). This trend was confirmed by using a generalized additive model (GAM) that modeled whether a call was part of a track with a logistic link function and the date and time of the call with both LOESS (LOcally Estimated Scatterplot Smoothing) and smoothing spline functions.

Localization precision can be estimated several different ways. In the time domain, the theoretical timing error is given by

$$\begin{array}{c} \Delta t=\frac{1}{\left(f_{2}-f_{1}\right)\sqrt{RL/NL}} \end{array}$$ (18)

(from [32]) where *f*_2_ − *f*_1_ is the bandwidth of the signal and RL/NL is the signal to noise ratio. We use 20–100 Hz for M3 cross-correlation or a bandwidth of 80 Hz. M3 calls within the hydrophone array have a median signal to noise ratio of about 10 dB (3.19). The timing error is therefore 7 ms. Using the assumed sound speed of 1500 m/s, the localization error is 10.5 m. However, we are cross-correlating calls in the spectrogram domain. The spectrogram resolution is 26 ms, which corresponds to a localization error of 38 m. Helble et al. [28] (Section IID) use Monte Carlo simulations to calculate the estimated timing error in humpback whale calls. For a single grunt humpback call in medium noise, the expected timing delay error is approximately 10 ms, which would correspond with a location error of 15 m. Propagation effects are not modeled in this method and could further increase the timing delay errors. Another way to estimate localization precision is to compare the call localizations with the “corrected” localizations based on the smoothing spline used to make tracks. We assume that the smoothing spline better models animal movement than straight lines connecting each successive localization. The mean of the median difference between the original localization and the smoothing spline “corrected” localization is 39 m with a standard deviation of 26 m. We therefore estimate that our call localizations have a precision of approximately 40 m.

#### Swimming behavior

**Fig 7:**
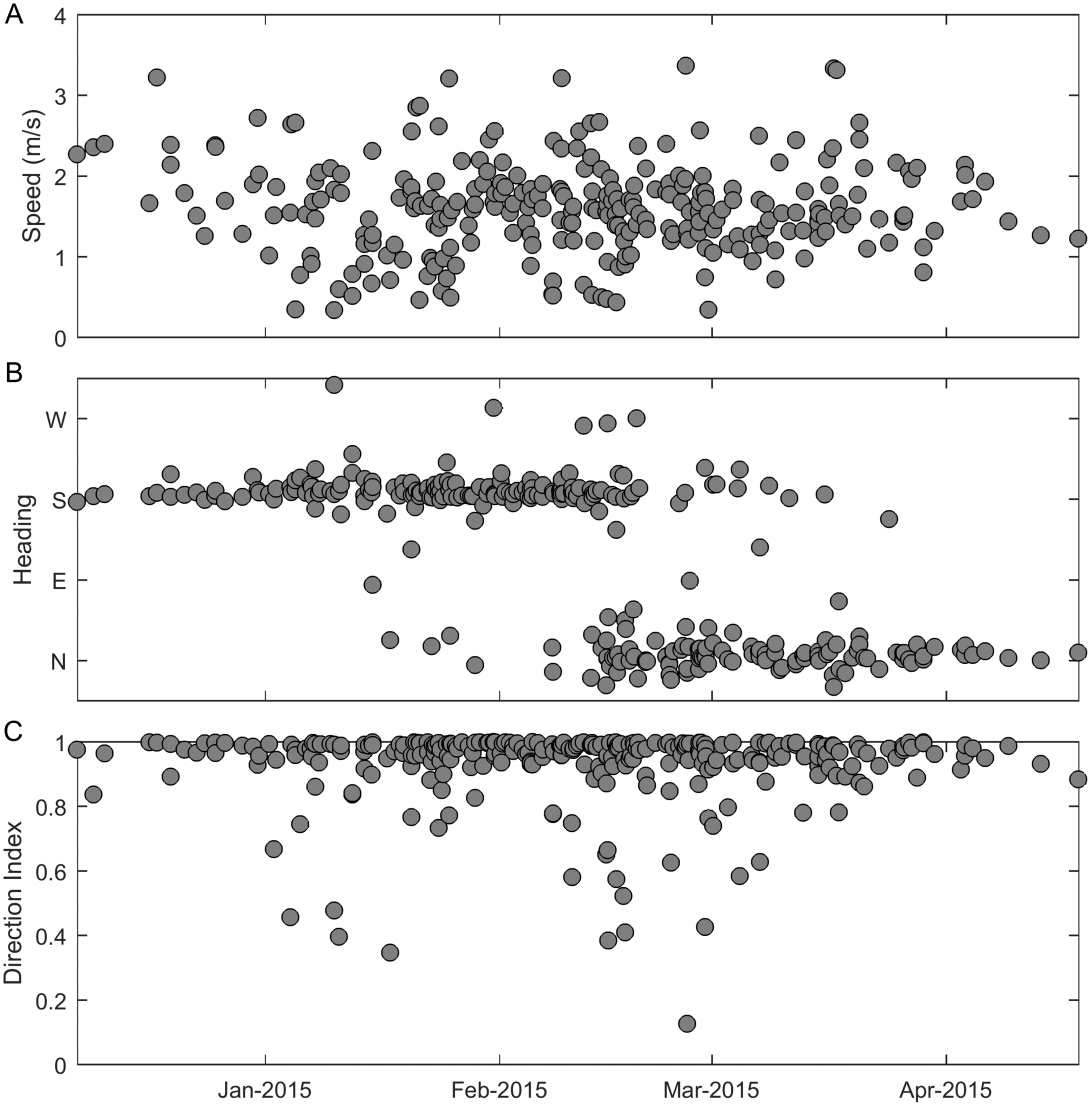
Seasonal cycle: Swimming behavior. Three track metrics used to assess swimming behavior of vocalizing gray whales. (A) displays average speed, (B) displays average heading, and (C) displays direction index.

Vocalizing whales swam with a mean speed of 1.6 m/s (standard deviation 0.59 m/s) (Fig 7A). No change in speed occurred over the migration season. Southbound whales dominated until the middle of February and then northbound whales became most prevalent (Fig 7B). The direction index shows that vocalizing gray whales are usually traveling along relatively direct paths, but may meander more in the first half of January and the second half of February into March (Fig 7C).

#### Seafloor depth

**Fig 8:**
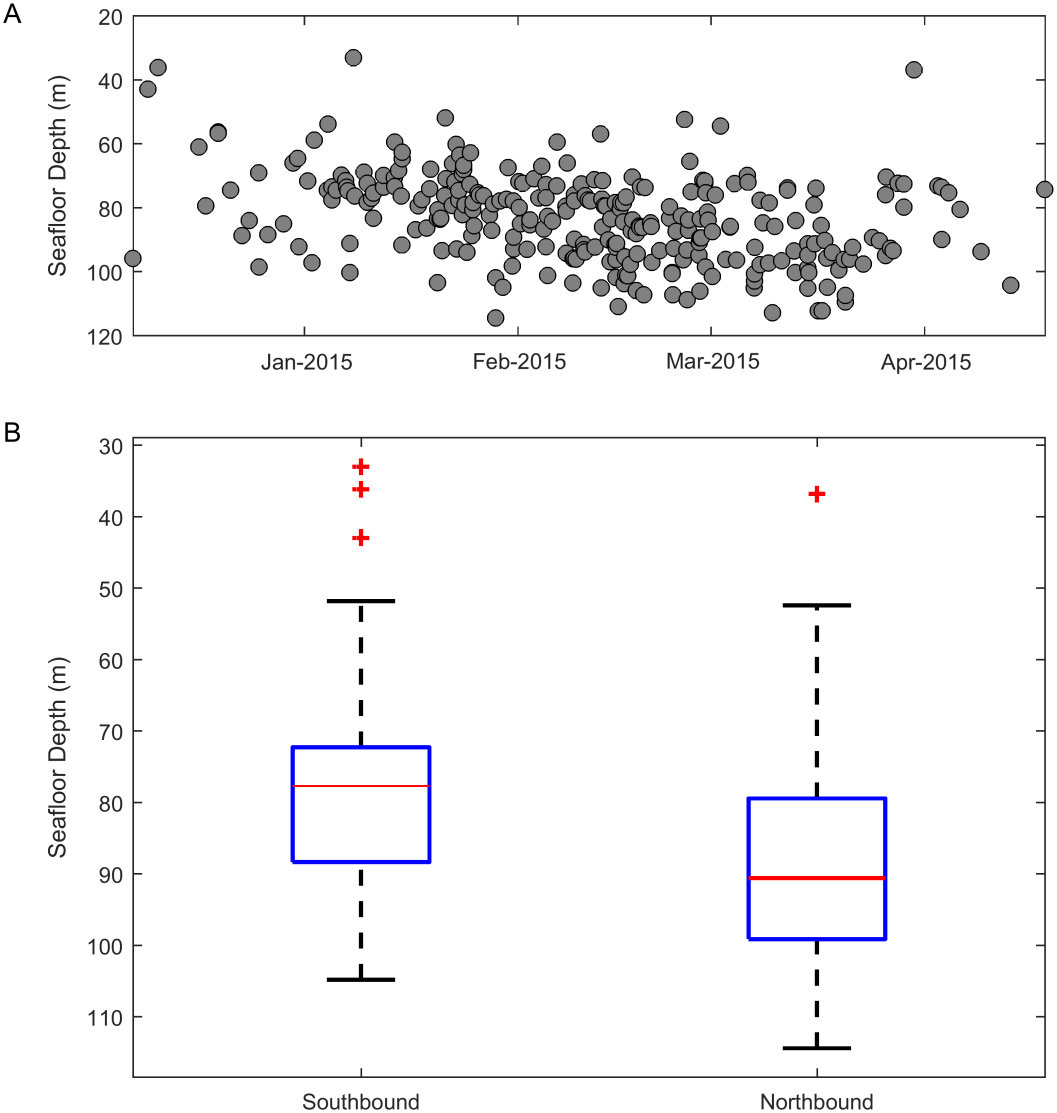
Seasonal cycle: Seafloor depth. The mean seafloor depth at the position of tracks over the migration season. (A) shows depth as a function of time and (B) categorizes tracks as southbound or northbound based on their heading and shows the same data in boxplot format.

The depth of water in which the tracks were located increased over the migration season (Fig 8). We used track heading to separate southbound and northbound migrators and found that northbound tracks were in significantly deeper water than southbound tracks (2-sample t-test, p = 7.0 × 10^−9^). The mean seafloor depth for southbound tracks was 79 m and the mean seafloor depth for northbound tracks was 89 m.

#### Average calling rate

A total of 4,247 gray whale M3 calls were localized within the bounds of the hydrophone array. From the probability of localization corrections described in detail above, we estimate that 4,854 calls were actually produced within the array. Using a distance of 2.28 km, a straight line distance approximating the 80 m bathymetric contour, and an average speed of 1.6 m/s, we estimated that it took 1.6 × 10^−2^ days (23.75 minutes) for a whale to swim through the array. Assuming that 90% of the 28,790 [14] whales migrated through the array and each whale traveled through during both the southbound and northbound migration, we estimated the calling rate as 5.7 calls/whale/day (0.24 calls/whale/hour).

### Diel cycle

#### Background noise and probability of localization

The mean background noise levels during the day (94.25 dB re 1 *μ*Pa root median square in the 20–100 Hz band) were not meaningfully different from the mean background noise levels during the night (94.09 dB re 1 *μ*Pa root median square in the 20–100 Hz band). In both cases, the probability of localization in the search area was close to 1. Therefore, diel calling differences cannot be explained by a change in noise level.

#### Total number of calls and percentage part of a track

**Fig 9:**
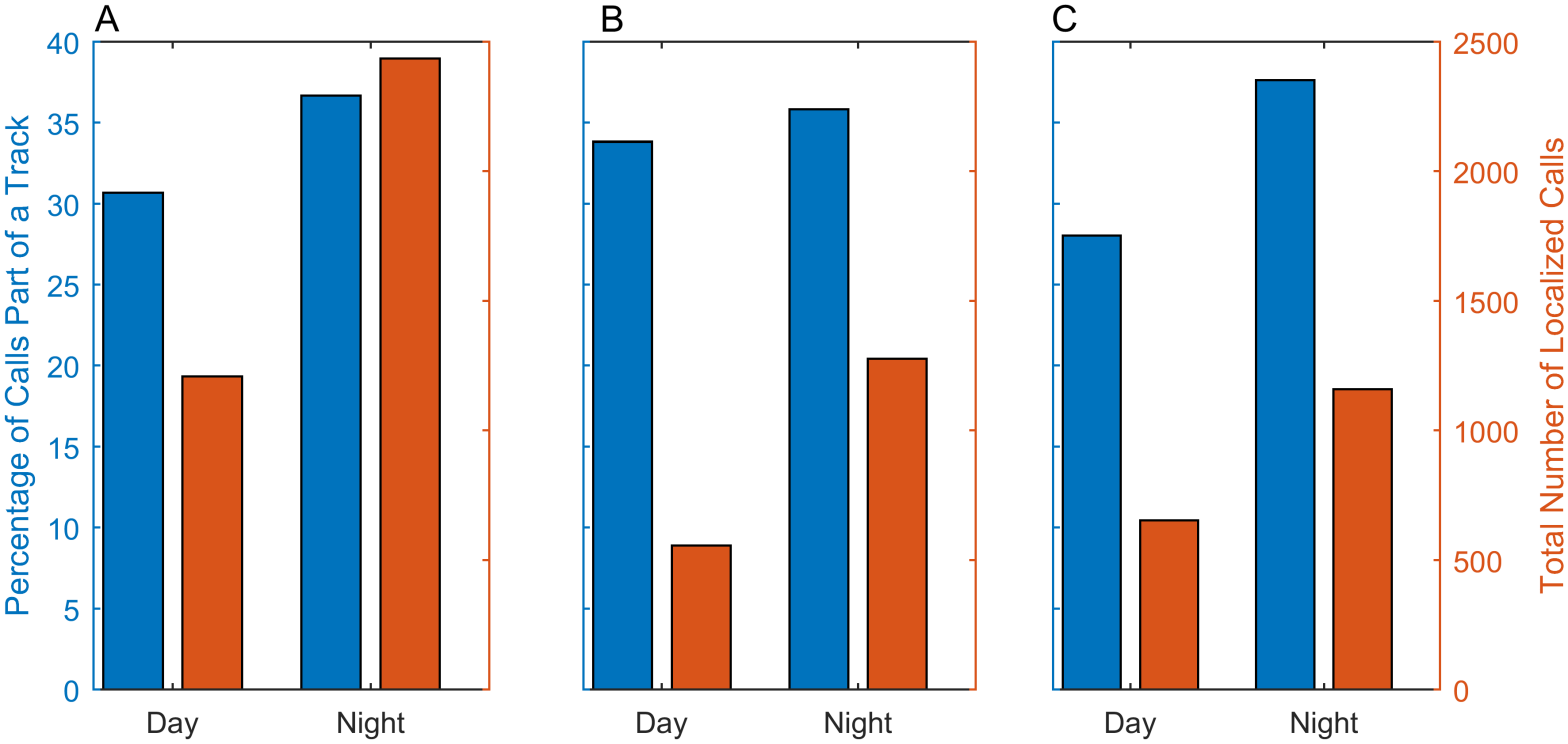
Diel cycle: Total calls and percentage of calls that were part of a track. The total number of localized calls (orange) compared with the percentage of those calls that were part of a track (blue). The calls are binned in daytime or nighttime according to the time they occurred with respect to the local sunrise and sunset. (A) shows the data for the full migration, (B) shows the data for calls before 15 February, and (C) shows the data for calls on or after 15 February. All of the calls included were manually verified to be gray whale M3 calls.

Over twice as many localized gray whale calls occurred during the night than during the day and the percentage of calls that were part of a track also increased (Fig 9A) (Fisher’s exact test, p = 3.4 × 10^−4^). In order to test if a statistically significant difference in diel calling between southbound and northbound migrants existed, we categorized all calls before 15 February as southbound and all calls on or after 15 February as northbound. Again, about twice as many calls occurred during the night than during the day for both halves of the migration. The percentage of calls that were part of a track was similar between day and night for the first half (Fisher’s exact test, p = 0.43) (Fig 9B), but the percentage of calls that were part of a track was greater at night during the second half (Fisher’s exact test, p = 3.6 × 10^−5^) (Fig 9C).

**Fig 10:**
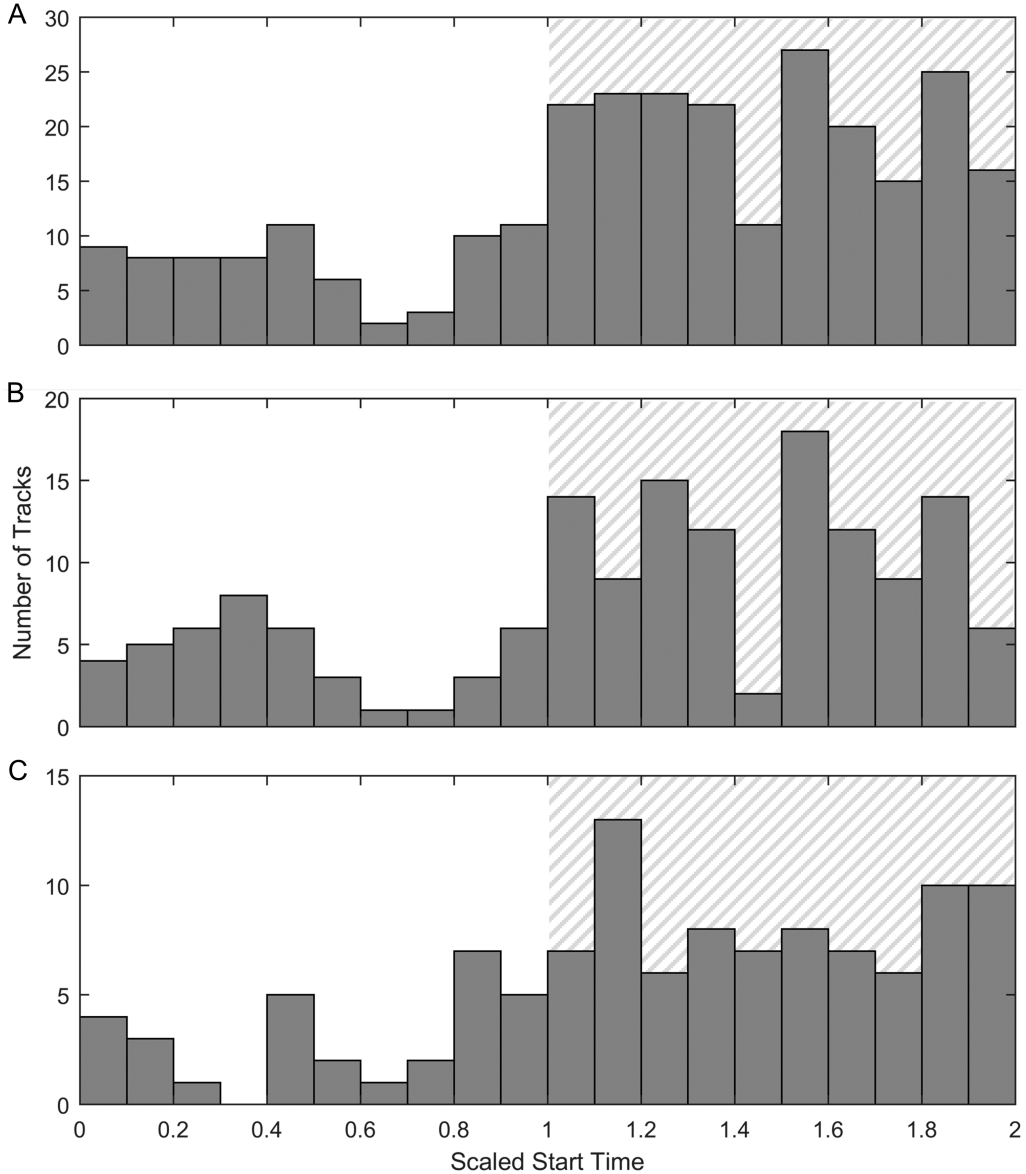
Diel cycle: Number of tracks. The total number of gray whale tracks binned into different start times. Since the time of sunrise and sunset changes substantially throughout the migration, these plots use scaled start time of each track where 0 indicates sunrise, 1 indicates sunset, and 2 indicates the following sunrise with gray hatching indicating night. (A) shows the entire migration, (B) shows all tracks with a southbound heading, and (C) shows all tracks with a northbound heading.

The change in calling over the diel cycle was further examined by looking at the number of tracks throughout the day. Since the time of sunrise and sunset varied throughout the migration, we used a scaled start time for each track and represented sunrise as 0, sunset as 1, and the following sunrise as 2. We compared the timing distribution of tracks to the distribution that would be expected if the timing of tracks within each day was random. Using a one-sided two-sample Kolmogorov-Smirnov test, we concluded that the distribution of calls was significantly different than a randomized distribution and that more tracks existed at night (p = 2.7 × 10^−7^ for the entire migration, p = 8.1 × 10^−4^ for southbound whales, and p = 1.3 × 10^−6^ for northbound whales) (Fig 10). Of the 280 gray whale tracks, 73 occurred entirely during the day, while 197 occurred entirely during the night (108/154 during the night versus 42/154 during the day for southbound whales, 78/112 during the night versus 28/112 during the day for nourthbound whales). In this case and for the rest of the results, since only calls that were part of a track are included, heading is a valid metric to determine the migration direction of the whale.

#### Swimming behavior

**Fig 11:**
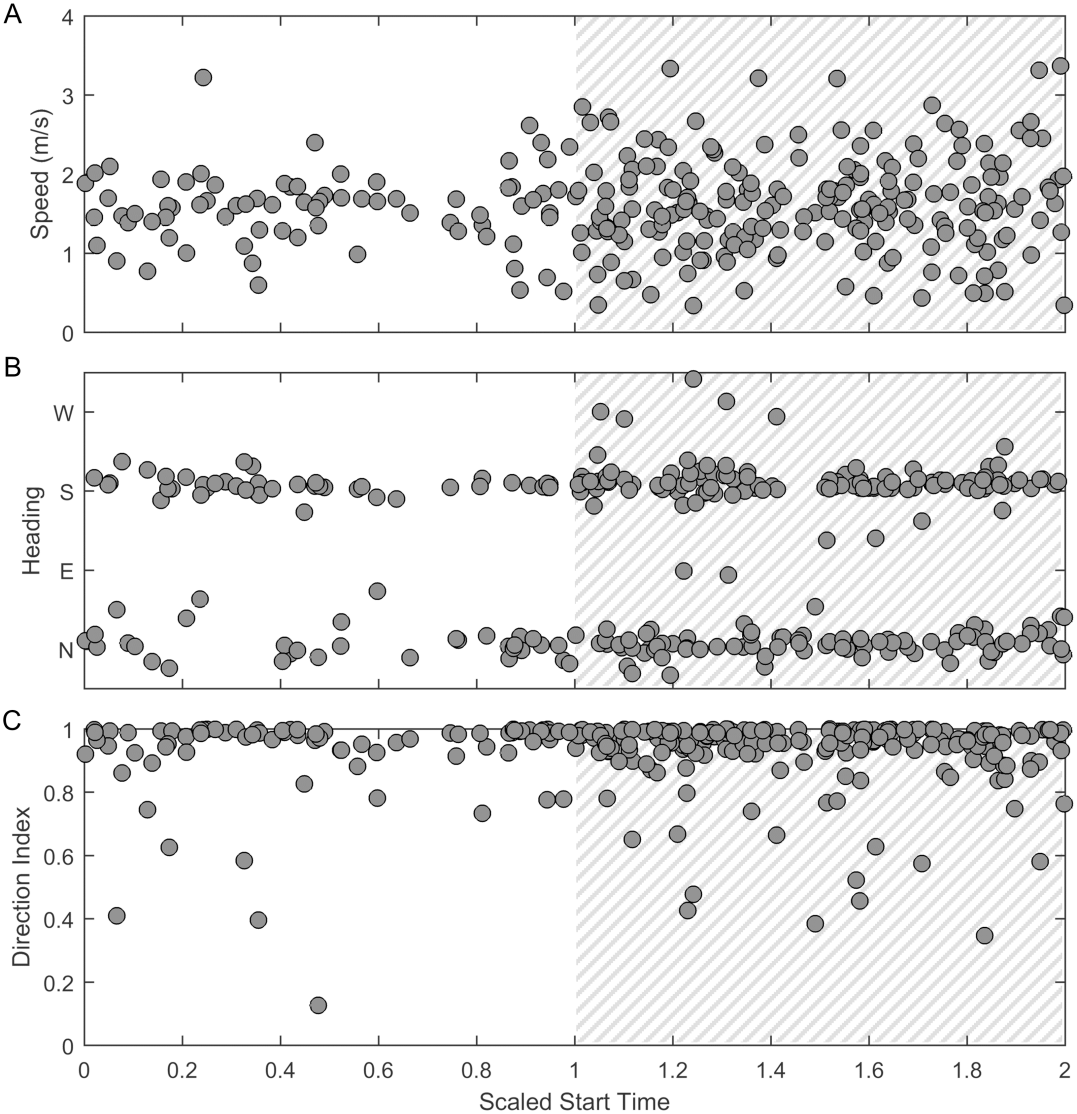
Diel cycle: Swimming behavior, entire migration. Three track metrics used to assess swimming behavior of vocalizing gray whales. (A) displays speed, (B) displays heading, and (C) displays direction index. All track metrics are shown as a function of scaled start time of the tracks where 0 indicates sunrise, 1 indicates sunset, and 2 indicates the following sunrise with gray hatching indicating night.

**Fig 12:**
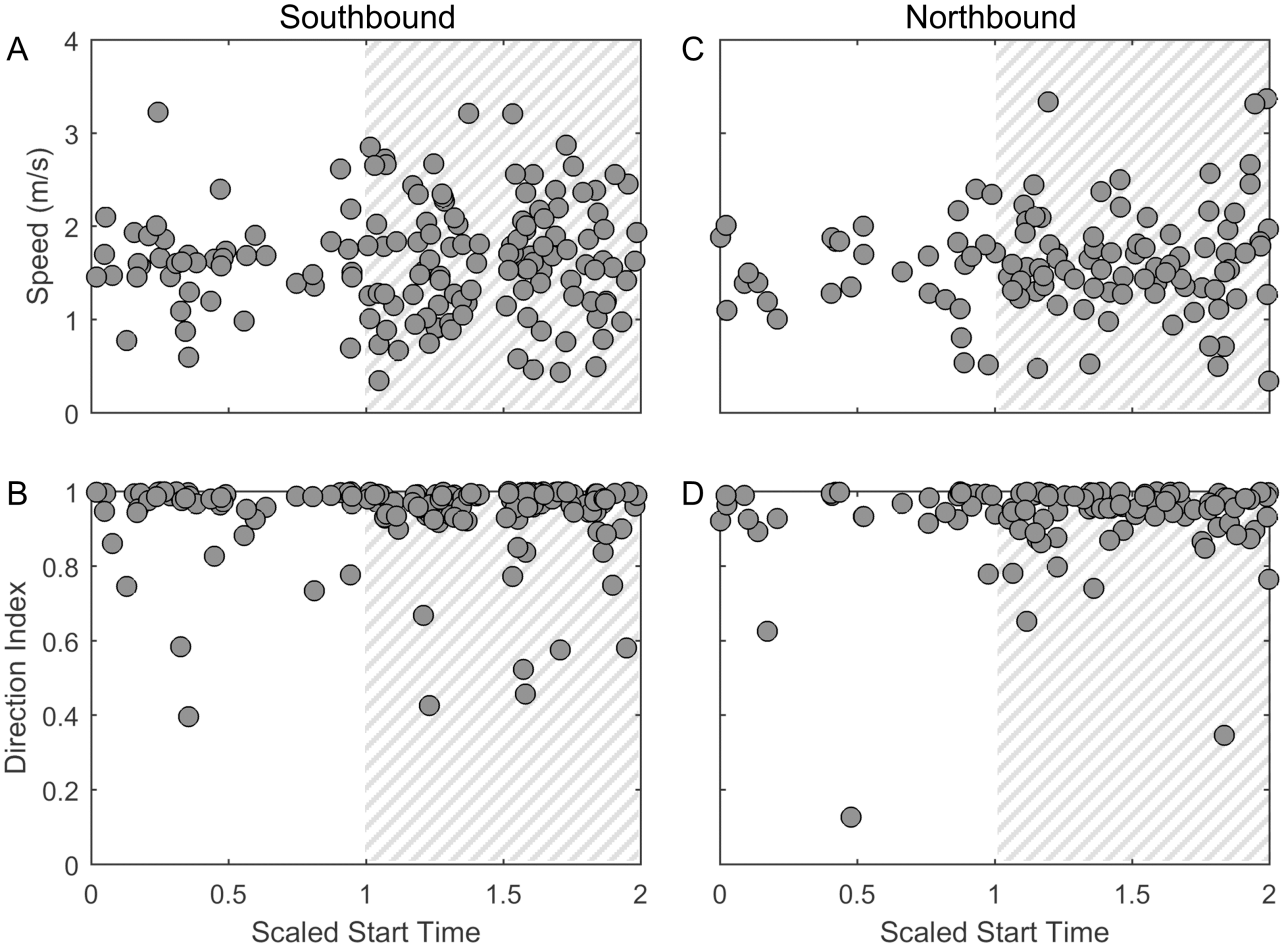
Diel cycle: Swimming behavior, split by heading. The speed (A and C) and direction index (B and D) split based on track heading. All track metrics are shown as a function of scaled start time of the tracks where 0 indicates sunrise, 1 indicates sunset, and 2 indicates the following sunrise with gray hatching indicating night.

The mean speed did not change in a statistically significant way over the diel cycle. This observation holds true when grouping all the tracks together from the entire migration (Fig 11) and when separating the tracks by southbound and northbound heading (Fig 12). However, the variance in speed was greater at night and this difference was significant for the entire migration and for whales swimming southbound (Levene’s test, p = 0.010 for the entire migration, p = 0.0044 for southbound whales). The direction of the tracks did not change between night and day.

#### Seafloor depth

**Fig 13:**
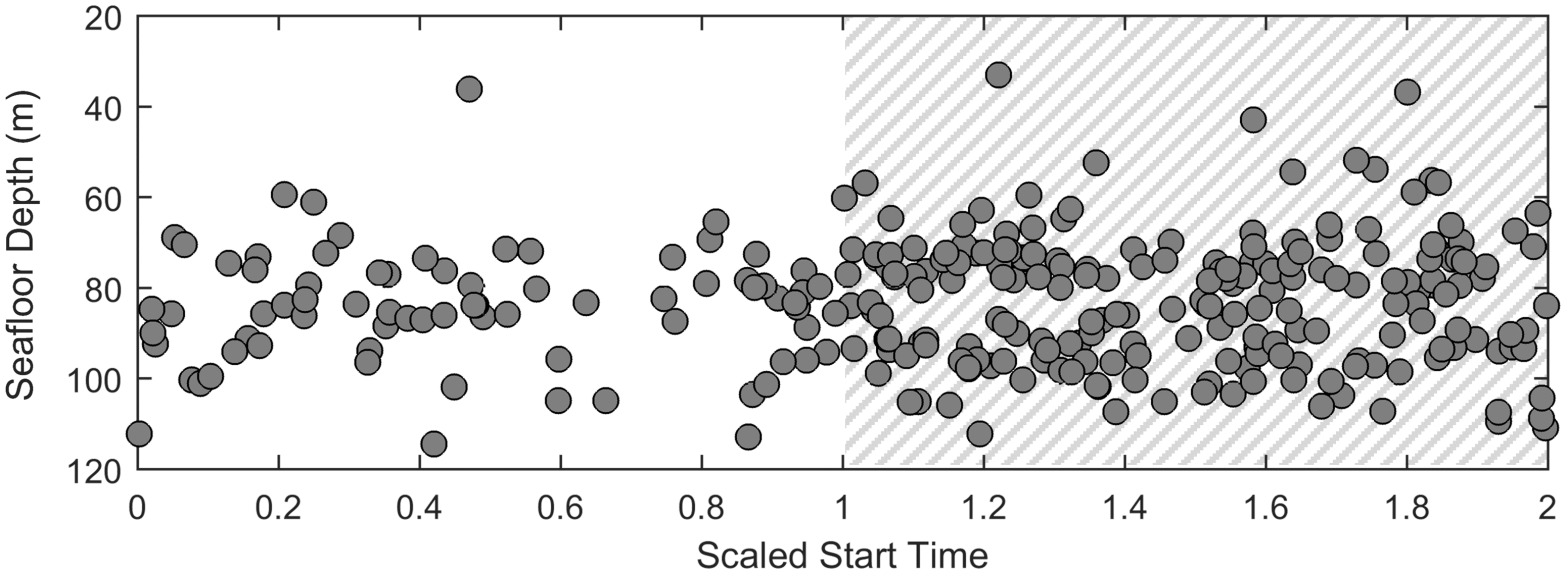
Diel cycle: Seafloor depth. The mean seafloor depth of tracks as a function of scaled start time where 0 indicates sunrise, 1 indicates sunset, and 2 indicates the following sunrise with gray hatching indicating night.

Tracks were in the same mean water depth during both night and day, however the variance at night was greater than the variance during the day (Levene’s test, p = 0.041) (Fig 13).

## Discussion

These results show that gray whales are acoustically active while migrating and have an average population calling rate that is about five times that previously reported on the migration route [23] and is approximately equal to the reported lagoon calling rate in March [22]. The calling rate is highly variable and those animals whose calls form tracks are calling much more frequently than the average calling rate. Other species of baleen whales have also been shown to have highly variable calling rates and tend to either be in a behavioral state in which they are vocalizing often or a behavioral state in which they are not vocalizing. For example, in a tagging study focused on North Atlantic right whales, over half of the individuals were silent for the duration of the tag recording and call rates ranged from 0 to 200 calls/hour [33]. The seasonal timing of the gray whale calls confirms the gray whale migration timing reported by visual observers. These findings suggest that gray whales change their swimming and acoustic behavior over seasonal and daily time scales.

This study describes the longest duration acoustic dataset focused on migrating gray whales with the greatest number of detected calls that has been published to date. This study is also the first to show full-season acoustic tracking of migrating gray whales. Using multiple hydrophones to localize calls is an effective method for reducing the false alarm rate of an automated detector because even though an automated detector may produce false detections in individual hydrophone sound files, the likelihood of noise detections at approximately the same time on all hydrophones is extremely low. Using multiple hydrophones allows for creating a known study area that can be monitored with a high probability of detection in nearly all noise conditions, unlike a single sensor where source locations are unknown and probability of detection changes with noise [30]. Acoustic localization and tracking is an important tool that can be applied to many regularly vocalizing species.

### Vocalization characteristics

Characteristics of the M1 and M3 calls were quantified and described as has been done in past studies. These results illustrate the importance of stating and understanding the methods to calculate each quantity. As is apparent in the mean values for each of the characteristics for the M3 and M1 call types, similar sounding metrics could be measuring very different aspects of the call. For M3 calls for example, the “peak frequency” is the frequency of the harmonic with the highest amplitude and is usually either the first or second harmonic, while the “mean frequency” is approximately the frequency of the second harmonic. The “3 dB bandwidth” is the bandwidth of the strongest harmonic, while the “±*σ* bandwidth” is the bandwidth of the entire received call. Confusion about call characteristics can lead to mis-categorization of call types. These mean values are helpful for identification of gray whale calls in other datasets, but it is imperative to note that these values are of the received call and not of the call produced by the whale and can be highly dependent on the environment. Constructive and destructive interference of multipaths will change the received waveform. High frequencies attenuate faster than low frequencies and in shallow water, sound cannot propagate at frequencies below the cutoff frequency of the first mode.

In this study, the variance in call characteristics due to the environment was separated from the total variance. The actual statistical separation of the variance due to the environment and the variance due to the calling animals themselves is limited by the sampling size of the recording sites. Increasing the number of recording sites to greater than four in order to provide greater variability in recording location would provide greater statistical separation. At the least, however, the variance of the characteristics of a given call across recording sites provides quantitative information on the impact of site-specific effects on localization. We would expect gray whales to use call properties that are robust to propagation to convey information. Again, propagation effects are apparent in the variance of a characteristic of a single call across the four sensors ($SD_{per\;call}^{2}$). The property most robust to environmental propagation (lowest percentage of total variance from variance per call) is mean frequency (23.8% of total variance is from variance per call). The other quantities are sensitive functions of the propagation characteristics and are good indicators of propagation variability but not good metrics for the call characteristics themselves. The same call could not be compared between hydrophones for M1 calls since few were detected with high SNR on all four hydrophones. Instead variation of inter-pulse interval was compared within a single call and between calls. Much of the variation in inter-pulse interval is due to differences between individual calls, but over one-third of the variation is from variability within a single call. Both the received M1 and M3 call types are highly variable. The best way to identify calls is to compare the general frequencies, duration, spacing between calls or pulses, and spectrogram contour shape with known gray whale calls. In addition, these quantities are affected by hydrophone sensitivities, so the frequency band monitored must be stated.

Using the hydrophone array setup, gray whale M3 call source levels were estimated. These source levels were in the range of those reported by Cummings et al. [17] from migrating whales and by Petrochenko et al. [24] from whales in the northern feeding areas. However, neither of these previously published results stated how source level was calculated (RMS, SEL, or peak-to-peak) or the bandwidth and only one publication stated the transmission loss assumptions. A 18.1 dB difference exists between the RMS estimate and peak-to-peak estimate, which is equal to a 10^1.81^ = 64.6 difference in pressure amplitudes squared, equivalent to the ratio of potential energy densities. Transmission loss assumptions affect the estimated source level. We assumed that the whale was calling near the surface and that neither source level nor attenuation and absorption were dependent on the location of the whale. If attenuation/absorption is dependent on specific call properties such as frequency content or location, then a call-specific *α* value should be used. Using a call-specific *α* value, the mean source levels are equivalent to those calculated using a mean *α* value within experimental accuracy (source level values are 0.1 dB greater with a call-specific *α*), however the source level values have higher variance (RMS *SD*^2^ is 53.5 dB when a call-specific *α* is used compared to 11.4 dB when a mean *α* is used). If the whales do not call near the surface, the estimated source level will change more significantly. For example, changing whether the whale vocalizes at the surface or at a depth of half the water depth would result in a change in transmission loss and therefore estimated source level of approximately 3 dB. For this analysis, the variation in the estimated source level due to variations in transmission loss can be estimated by the variance for the same call across all four hydrophones. The standard deviation of the means for M3 call source levels was just over than 3 dB. The transmission loss could be expected to vary on this order if the animals call over a wide interval of depths rather than close to a single depth. In addition, since the environmental properties are not homogenous across the survey area due to the sloping bottom and possibly other characteristics, the attenuation and absorption will be different even for a single call as it travels to each of the four hydrophones. This simple spherical and cylindrical spreading model does not take these propagation differences into account, but the variation in source level due to differences propagating to each of the hydrophones for a single call is quantified in the mean of the variances for a single call. Further, the reported source levels may be slightly in error due to full wavefield propagation effects such as the Lloyd’s mirror effect and the different excitation of modes at the depths of the whale compared to the depths of the hydrophones. We have reduced some of these impacts by averaging the results from four hydrophones at different depths and locations.

### Seasonal cycle

The fidelity of the direction of migration and its dependence on season was quantitatively evaluated, as well as other metrics of the migration paths. Most of the acoustically tracked southbound whales passed Granite Canyon between the beginning of December and mid-February with the steadiest stream of tracks during January and the first half of February. This result matches the migratory timing reported by visual surveys.

Although there exists high variability in the percentage of calls that were part of a track over the entire migration season, the increase in percentage of calls that were part of a track at the beginning and end of the migration (Fig 5) could indicate a change in how vocalizations are used by different demographics of whales. Pregnant females are the first to migrate south and postpartum females with calves are the last to migrate north [3]. The beginning and end of the migration season is marked by the least total number of gray whale calls, but the highest percentage of calls that were part of a track. We hypothesize that the pregnant females and those same females with their calves may call more often than other whales as they migrate, which makes their calls more likely to form a track, but the number of whales is more sparse, resulting in a lower total call count. This difference in behavior may be because these females are usually traveling alone, or at least without another mature whale, and are calling to keep in contact with more distant whales.

The gray whale swimming behavior results obtained from acoustic tracking confirm many of the results reported by previous studies. Using acoustic tracking allowed us to monitor for an entire migration cycle and obtain a sample size of 280 tracks which is larger than those of previous behavior studies that used tagging and visual methods. The mean speed of the acoustic gray whale tracks was 1.6 m/s, which is near the middle of the range reported by previous publications [1, 3, 17–21]. Most of the tracks were very direct supporting the idea that the whales are primarily migrating to their destination and do not deviate to engage in other behaviors. The meandering tracks in the middle of the migration season may be examples of the social and sexual behavior that visual observers have noted at similar times in other years [8, 19]. The tracks were slightly farther offshore in deeper water during the northbound migration than the southbound migration. This shift was not an extreme difference, which could be in part because of the narrow shelf in the study area, but the shift to deeper water agrees with previous observations that most whales travel north farther offshore, perhaps to get to their feeding areas more quickly [4]. Females with calves migrate north very nearshore, but calves make up a very small percentage of the entire population [5, 6]. In addition, the very nearshore sounds of breaking waves create an acoustic environment with an unknown probability of detection, so we did not attempt to track gray whales in or near the surf zone. Acoustic masking in kelp beds has been suggested by others as a way for gray whales to avoid predation from killer whales (*Orcinus orca*) [4].

### Diel cycle

Gray whale behavior within the study area changed between night and day. Most significantly, an increase in calls detected occurred at night even though the probability of detection did not change. An increase in nighttime calling has also been reported in several other mysticete species such as humpback whales, blue whales, and North Pacific right whales [34–36]. In addition, an increase occurred in the percentage of calls that were part of a track at night. We hypothesize that gray whales may call more often when they can no longer see their nearest neighbor.

One assumption of population size estimates is that gray whales are increasing their southbound migration rate at night [15]. In contrast to Perryman et al. [19], we did not observe a change in mean speed over daily or seasonal time scales. Speed variance did increase at night however. If gray whales are not changing their mean migration speed between night and day, this result would warrant a change in how daytime visual counts are extrapolated to the night which would result in a population size that is lower than reported.

Similar to speed, mean water depth over which the tracks were located was the same at night and day but depth variance increased at night. If gray whales are using visual cues of land or the seafloor to aid their navigation, we would expect less direct tracks at night indicated by a decrease in direction index. In contrast, the direction index of tracks remained the same at night and day. We speculate that since more calling occurs at night, more individuals are producing sounds and these individuals have a wider variance in swimming behavior than the individuals that are calling during both the day and night. However, even though these individuals show a wider range of migration speeds and distance offshore, they still have the same migration goal and therefore their direction index is about the same.

This research was limited in that we were sampling at one location, during one migration cycle, and we are only able to track vocalizing animals. Future studies should investigate whether gray whale behavior changes at different locations along the migration, from year to year, or between vocalizing and non-vocalizing animals.

## Conclusion

The recordings of a set of marine mammal calls by three or more receivers allows both for 1) localization and potential tracking of a calling animal, and 2) separation of the total variance of the calls into a component associated with environmental variability and a component associated with the calling animals themselves. In order to quantify the effects of environment-specific propagation characteristics, no additional numerical modeling or signal processing is required.

Acoustic localization and tracking of animals deepens our understanding of behavior that is difficult or impossible to observe visually. For example, we determined that gray whales increase calling at night and call more regularly toward the beginning and end of the migration season. These results provide clues as to the utility of calls for the gray whale migration. In addition, we observed that vocalizing gray whales swim at the same average speed at night and day. This finding challenges an assumption that is used in population size calculations based on visual surveys.

In the past, researchers have relied on categorization of calls using measured characteristics. In this study, multiple recordings of the same call on separate hydrophones demonstrate that received call characteristics can be highly variable and are dependent on both the animal producing the sound and the local propagation effects. These values can be helpful for initial identification of potential calls, but the variability due to environmental effects must be appreciated. Moreover, different methods of calculating source levels and other call characteristics significantly change the resulting quantities, so detailed methods and assumptions should always be stated so that results can be compared to the analyses of other datasets.

Using multiple hydrophones in close proximity to study marine mammals can greatly increase our knowledge about both acoustic and swimming behavior. This methodology has the potential to allow us to quantify aspects of behavior that are actually due to modification of produced signals within the environment and determine the true behaviors of the animals.

## Supporting information

S1 Fig Acoustic recording package mooring diagram. This diagram shows the design of the bottom-moored acoustic recording package. The hydrophones are located 15 m above the seafloor. The green circles indicate the locations of the floats. The data logger contains the batteries, computer, and hard drives. The release is an acoustic release system that is used to retrieve the package along with the data at the end of the deployment. This diagram is not to scale.
(TIF)

S1 Audio Sound file of a gray whale M3 call. This audio file is the same M3 call pictured in the spectrogram and time series in Fig 2. It was recorded on the NE Granite Canyon hydrophone.
(WAV)

S2 Audio Sound file of a gray whale M1 call. This audio file is the same M1 call pictured in the spectrogram and time series in Fig 2. It was recorded on the NE Granite Canyon hydrophone.
(WAV)

## References

[1] Sumich JL. Swimming velocities, breathing patterns, and estimated costs of locomotion in migrating gray whales, *Eschrichtius robustus*. Canadian Journal of Zoology. 1983;61(3):647–652. doi: 10.1139/z83-086

[2] Rugh DJ. Census of Gray Whales at Unimak Pass, Alaska, November–December 1977–1979 In: Jones ML, Swartz SL, Leatherwood S, editors. The Gray Whale: *Eschrichtius robustus*. Orlando, FL: Academic Press; 1984 p. 225–248.

[3] Rice DW, Wolman AA. The life history and ecology of the gray whale (*Eschrichtius robustus*). The American Society of Mammalogists; 1971.

[4] Poole MM. Migration corridors of gray whales along the central California coast, 1980–1982 In: Jones ML, Swartz SL, Leatherwood S, editors. The Gray Whale: *Eschrichtius robustus*. Orlando, FL: Academic Press; 1984 p. 389–407.

[5] Perryman WL, Donahue MA, Perkins PC, Reilly SB. Gray whale calf production 1994–2000: Are observed fluctuations related to changes in seasonal ice cover? Marine Mammal Science. 2002;18(1):121–144. doi: 10.1111/j.1748-7692.2002.tb01023.x

[6] Perryman WL, Reilly SB, Rowlett RA. Results of surveys of northbound gray whale calves 2001–2009 and examination of the full sixteen year series of estimates from the Piedras Blancas Light Station; 2010. Paper SC/62/BRG1 presented to the International Whaling Commission Scientific Committee. Available from: http://iwc.int/.

[7] Sumich JL. *E. robustus*: The biology and human history of gray whales. Corvallis, OR: Whale Cove Marine Education; 2014.

[8] Gilmore RM. A census of the California gray whale. No. 342 in Special Scientific Reports: Fisheries. Washington, D.C.: US Department of Interior, Fish and Wildlife Service; 1960. p. iv–30.

[9] Shelden KEW, Laake JL. Comparison of the offshore distribution of southbound migrating gray whales from aerial survey data collected off Granite Canyon, California, 1979–96. Journal of Cetacean Research and Management. 2002;4(1):53–56.

[10] Scammon CM. The marine mammals of the north-western coast of North America, described and illustrated: Together with an account of the American whale-fishery. San Francisco, CA: John H. Carmany and Company; 1874.

[11] Hubbs CL, Hubbs LC. Gray whale censuses by airplane in Mexico. California Fish and Game. 1967;53(1):23–27.

[12] Moore SE, Urbán-Ramirez J, Perryman WL, Gulland F, Perez-Cortes M H, Wade PR, et al Are gray whales hitting “K” hard? Marine Mammal Science. 2001;17(4):954–958. doi: 10.1111/j.1748-7692.2001.tb01310.x

[13] Reilly SB, Bannister JL, Best PB, Brown M, Brownell RL Jr, Butterworth DS, et al Eschrichtius robustus. The IUCN Red List of Threatened Species. 2008;2008(e.T8097A12885255).

[14] Durban JW, Weller DW, Perryman WL. Gray whale abundance estimates from shore-based counts off California in 2014/15 and 2015/16; 2017. Paper SC/A17/GW/06 presented to the International Whaling Commission Scientific Committee. Available from: http://iwc.int/.

[15] Laake JL, Punt A, Hobbs RC, Ferguson M, Rugh DJ, Breiwick JM. Re-analysis of gray whale southbound migration surveys, 1967–2006. U.S. Department of Commerce; 2009. NMFS-AFSC203.

[16] Rugh DJ, Muto MM, Hobbs RC, Lerczak JA. An assessment of shore-based counts of gray whales. Marine Mammal Science. 2008;24(4):864–880.

[17] Cummings WC, Thompson PO, Cook R. Underwater sounds of migrating gray whales, *Eschrichtius glaucus* (Cope). The Journal of the Acoustical Society of America. 1968;44(5):1278–1281. doi: 10.1121/1.1911259 5699032 10.1121/1.1911259

[18] Rugh DJ, Ferrero RC, Dahlheim ME. Inter-observer count discrepancies in a shore-based census of gray whales (*Eschrichtius robustus*). Marine Mammal Science. 1990;6(2):109–120. doi: 10.1111/j.1748-7692.1990.tb00233.x

[19] Perryman WL, Donahue MA, Laake JL, Martin TE. Diel variation in migration rates of eastern Pacific gray whales measured with thermal imaging sensors. Marine Mammal Science. 1999;15(2):426–445. doi: 10.1111/j.1748-7692.1999.tb00811.x

[20] Mate BR, Urbán-Ramirez J. A note on the route and speed of a gray whale on its northern migration from Mexico to central California, tracked by satellite-monitored radio tag. Journal of Cetacean Research and Management. 2003;5(2):155–158.

[21] Mate B, Lagerquist B, Irvine L. Feeding habitats, migration, and winter reproductive range movements derived from satellite-monitored radio tags on eastern North Pacific gray whales. International Whaling Commission Scientific Committee; 2010. SC/62/BRG21.

[22] Dalheim ME. Bioacoustics of the gray whale (*Eschrichtius robustus*). University of British Columbia; 1987.

[23] Crane NL, Lashkari K. Sound production of gray whales, *Eschrichtius robustus*, along their migration route: a new approach to signal analysis. The Journal of the Acoustical Society of America. 1996;100(3):1878–1886. doi: 10.1121/1.416006 8817910 10.1121/1.416006

[24] Petrochenko SP, Potapov AS, Pryadko VV, Wood JS. Sounds, source levels, and behavior of gray whales in the Chukotskoe Sea. Soviet Physics Acoustics. 1991;37(6):622–624.

[25] Fish JF, Sumich JL, Lingle GL. Sounds produced by the gray whale, *Eschrichtius robustus*. Marine Fisheries Review. 1974;36(4):38–45.

[26] Wiggins SM, Hildebrand JA. High-frequency Acoustic Recording Package (HARP) for broad-band, long-term marine mammal monitoring. In: 2007 Symposium on Underwater Technology and Workshop on Scientific Use of Submarine Cables and Related Technologies. IEEE; 2007. p. 551–557.

[27] Helble TA, Ierley GR, D’Spain GL, Roch MA, Hildebrand JA. A generalized power-law detection algorithm for humpback whale vocalizations. The Journal of the Acoustical Society of America. 2012;131(4):2682–2699. doi: 10.1121/1.3685790 22501048 10.1121/1.3685790

[28] Helble TA, Ierley GR, D’Spain GL, Martin SW. Automated acoustic localization and call association for vocalizing humpback whales on the Navy’s Pacific Missile Range Facility. The Journal of the Acoustical Society of America. 2015;137(1):11–21. doi: 10.1121/1.4904505 25618034 10.1121/1.4904505

[29] Urick RJ. Principles of underwater sound for engineers. Tata McGraw-Hill Education; 1967.

[30] Helble TA, D’Spain GL, Hildebrand JA, Campbell GS, Campbell RL, Heaney KD. Site specific probability of passive acoustic detection of humpback whale calls from single fixed hydrophones. The Journal of the Acoustical Society of America. 2013;134(3):2556–2570. doi: 10.1121/1.4816581 23968053 10.1121/1.4816581

[31] Au WWL, Pack AA, Lammers MO, Herman LM, Deakos MH, Andrews K. Acoustic properties of humpback whale songs. The Journal of the Acoustical Society of America. 2006;120(2):1103–1110. doi: 10.1121/1.2211547 16938996 10.1121/1.2211547

[32] Woodward PM. Probability and Information Theory, with Applications to Radar vol. 3 of International Series of Monographs on Electronics and Instrumentation. 2nd ed Fry DW, Higinbotham W, editors. Pergamon Press; 1964.

[33] Parks SE, Johnson M, Nowacek D, Tyack PL. Individual right whales call louder in increased environmental noise. Biology Letters. 2011;7(1):33–35. doi: 10.1098/rsbl.2010.0451 20610418 10.1098/rsbl.2010.0451 PMC3030867

[34] Au WWL, Mobley J, Burgess WC, Lammers MO, Nachtigall PE. Seasonal and diurnal trends of chorusing humpback whales winteing off western Maui. Marine Mammal Science. 2000;16(3):530–544. doi: 10.1111/j.1748-7692.2000.tb00949.x

[35] Wiggins SM, Oleson EM, McDonald MA, Hildebrand JA. Blue Whale (*Balaenoptera musculus*) Diel Call Patterns Offshore of Southern California. Aquatic Mammals. 2005;31(2):161–168. doi: 10.1578/AM.31.2.2005.161

[36] Munger LM, Wiggins SM, Moore SE, Hildebrand JA. North Pacific right whale (*Eubalaena japonica*) seasonal and diel calling patterns from long-term acoustic recordings in the southeastern Bering Sea, 2000–2006. Marine Mammal Science. 2008; doi: 10.1111/j.1748-7692.2008.00219.x

