# Supplementary figures and images for: Migratory behavior of eastern North Pacific gray whales tracked using a hydrophone array
Source: PLoS One. 2017 Oct 30;12(10):e0185585. doi: 10.1371/journal.pone.0185585 (PMC5662093; doi:10.1371/journal.pone.0185585)

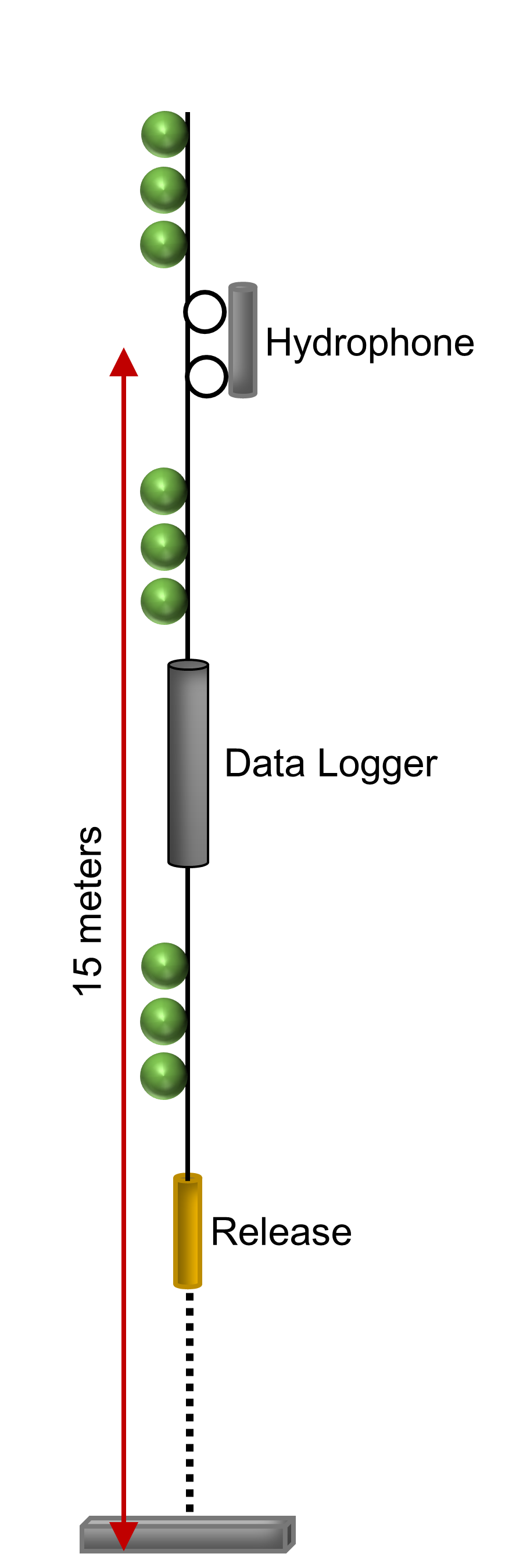

Supplement: S1 Fig — This diagram shows the design of the bottom-moored acoustic recording package. The hydrophones are located 15 m above the seafloor. The green circles indicate the locations of the floats. The data logger contains the batteries, computer, and hard drives. The release is an acoustic release system that is used to retrieve the package along with the data at the end of the deployment. This diagram is not to scale. (TIF) [file pone.0185585.s001.tif]
